# Validating simulated patient programmes in Obstetrics and Gynaecology education: a mixed-method study on training effectiveness and stakeholder perceptions in the GCC

**DOI:** 10.1186/s12909-025-07912-2

**Published:** 2025-10-17

**Authors:** Archana Prabu Kumar, Diaa Rizk, Zainab Al Jufairi, Taysir Garadah, Amal A. K. Alsubaiei, Hany Atwa, Mohamed Hany Shehata, Ahmed Al-Ansari, Abdelhalim Deifalla

**Affiliations:** 1 Medical Education Department, College of Medicine and Health Sciences (CMHS), Arabian Gulf University (AGU), Manama, Bahrain; 2 Department of Obstetrics & Gynaecology, CMHS, AGU, Manama, Bahrain; 3 Medical Skills and Simulation Centre (MSSC), CMHS, AGU, Manama, Bahrain; 4 Medical Student, CMHS, AGU, Manama, Bahrain; 5 Department of Family and Community Medicine, CMHS, AGU, Manama, Bahrain; 6 Anatomy Department, CMHS, AGU, Manama, Bahrain; 7 Family Medicine Department, Faculty of Medicine, Helwan University, Cairo, Egypt; 8 Medical Education Department, Faculty of Medicine, Suez Canal University, Ismailia, Egypt; 9 Chief Executive Officer, National Health Regulatory Authority (NHRA), Manama, Bahrain; 10 Human Anatomy and Embryology Department, Faculty of Medicine, Suez Canal University, Ismailia, Egypt

**Keywords:** Simulated patients, Medical education, History-taking, Obstetrics and gynaecology, Reproductive health, SP training, SP validation, Competency-based education, Inter-rater reliability, Cross-cultural communication

## Abstract

**Background:**

Simulated patients (SPs) are widely used in medical education to help students acquire clinical skills in a safe and realistic setting. In fields such as obstetrics and gynaecology (OB-GYN), where real-patient interactions can be limited due to cultural sensitivities—especially in Gulf Cooperation Council (GCC) countries—SPs play a crucial role in medical education. This study aimed to validate the training of SPs for assessing OB-GYN history-taking skills and explore the perceptions of stakeholders – including faculty, students, leadership and SPs – regarding the implementation, challenges and potential improvements of the SP programme.

**Methods:**

A mixed-methods research design was employed in this study. During the quantitative phase, training of SPs using a structured OB-GYN history-taking module was conducted, followed by an evaluation of their performance by expert raters. The inter-rater reliability was assessed using Fleiss’ Kappa and Cronbach’s alpha. In the qualitative phase, semi-structured interviews and focus groups were conducted with students, SPs, faculty trainers and academic leaders. Thematic analysis was performed to identify common themes and divergent viewpoints.

**Results:**

Quantitative results showed moderate-to-high inter-rater reliability, indicating consistent and effective SP performance post-training. Qualitative findings revealed that stakeholders perceived that the usage of SPs results in the enhancement of student engagement, realism and communication skills. However, challenges such as cultural sensitivities, limited SP diversity and logistical constraints were highlighted. Stakeholders emphasised the need for culturally contextualised training, periodic feedback loops and institutional support.

**Conclusions:**

The study confirms that structured training can effectively prepare simulated patients for OB-GYN education in GCC settings. By integrating stakeholder feedback, this study provides valuable insights for refining SP programmes and addressing region-specific challenges. A more culturally aware and well-supported SP programme can boost student confidence and improve learning outcomes in sensitive clinical areas like OB-GYN.

**Supplementary Information:**

The online version contains supplementary material available at 10.1186/s12909-025-07912-2.

## Background

History-taking plays a fundamental role in gathering accurate patient information and serves as the basis for effective doctor–patient communication, diagnosis and patient safety [1, 2]. In obstetrics and gynaecology (OB-GYN), this process involves obtaining information about the reproductive health and sexual practices of the patient [3, 4]. Discussing such information is often challenging, particularly in cultures where open conversations about reproductive health are uncommon, and it may be even more difficult when patients interact with a healthcare provider of the opposite gender [5, 6].

Cultural awareness and the ability to adapt to diverse patient backgrounds—including those with past trauma or specific sensitivities—are crucial in gathering accurate medical histories [7]. However, in Arab societies, cultural norms often discourage women from openly expressing personal concerns. The intimate and sensitive nature of obstetric and gynaecological diseases in a predominantly strict and conservative socio-religious environment significantly influences a preference for same-gender physician–patient dyads [8]. Most women prefer consulting female OB-GYN doctors due to a combination of factors, including discomfort during pelvic examinations, religious beliefs, cultural norms that emphasise modesty and broader cultural practices regarding gender interaction [9]. These internalised customs can lead to discomfort when discussing intimate health concerns or undergoing pelvic examinations with male healthcare providers. Consequently, this affects the participation of male medical students and residents in OB-GYN clinical care, thus limiting their opportunities for experiential learning and hands-on skill acquisition. This sociocultural barrier has significant implications for undergraduate and postgraduate medical education and the delivery of OB-GYN services [9].

Cultural differences significantly shape how sexual health is perceived and addressed in clinical practice. For instance, physicians from Western, Asian and Middle Eastern backgrounds often experience varying degrees of taboo and discomfort when discussing sexual health, influenced by their cultural upbringing [10]. These cultural barriers can result in inadequate patient communication, underreporting of sexual issues and missed opportunities for intervention. Thus, addressing such cultural differences is essential for delivering effective and equitable sexual health care [10]. For practitioners working in the Middle East, understanding these dynamics is essential for providing culturally competent care and improving patient trust and health outcomes [11]. SP training in OB-GYN equips students with the skills needed to navigate these cultural sensitivities, ensuring they can communicate effectively while respecting patient boundaries [6, 12, 13].

While there is a growing recognition of the importance of simulation-based training and communication skills training in medical education, the implementation of structured SP training specifically for OB-GYN history-taking is still in its early stages [14]. While SPs are widely used for history-taking assessments in other medical fields, little research validates their reliability and effectiveness in OB-GYN education in Middle Eastern medical institutions. By addressing this gap, this study aims to develop and validate a culturally adapted simulated patient (SP) training programme for OB-GYN history-taking in a Middle Eastern medical college. By identifying strategies for navigating cultural barriers, this research will contribute to the effective implementation of SP-based training in OB-GYN education in conservative settings.

Furthermore, the integration of SP programmes in GCC countries must contend with region-specific complexities such as cultural values, language diversity, gender norms and healthcare system structures that significantly shape the design and delivery of medical training [15–18]. These variables can influence learner engagement, teaching strategies, assessment methods and feedback processes. Therefore, to ensure meaningful and culturally congruent simulation experiences, it is essential to understand how SP programmes are perceived by all stakeholders involved, including students, faculty trainers, institutional leaders and the SPs themselves. Given this evolving yet under-explored educational landscape, a deeper understanding of stakeholder perspectives is crucial to inform culturally sensitive and sustainable SP programme development.

Kolb’s Experiential Learning Theory (ELT) [19] was chosen as the theoretical framework for this study. This theory aligns well with the structure of SP programmes in which learners actively engage in clinical encounters (concrete experience), reflect on their performance through feedback (reflective observation), integrate feedback and theoretical knowledge (abstract conceptualisation), and then apply these insights in subsequent interactions (active experimentation). Applying Kolb’s model allowed us to frame the SP encounters not merely as practice opportunities but also as structured pedagogical tools for deeper learning, especially in sensitive areas like OB-GYN.

This mixed-methods study aims to evaluate and contextualise the design, implementation and effectiveness of a culturally responsive SP training programme for OB-GYN history-taking in a medical college in the Middle East. The study combines the quantitative assessment of the effectiveness of simulated patients with a qualitative exploration of stakeholder perspectives. Specifically, it investigates the perceived value, implementation challenges and suggestions for improvement. By focusing on the voices of individuals directly engaged in this programme, the present research seeks to provide culturally responsive, practice-oriented insights that can inform the development of future curriculum and educational policy in health professions in comparable settings.

### Objectives

1. To design and implement a structured training programme for simulated patients (SPs) tailored towards OB-GYN history-taking, aligning with medical education standards and regional cultural sensitivities.
2. To evaluate the effectiveness and reliability of trained SPs in simulating OB-GYN scenarios and assessing medical students’ clinical history-taking skills using validated performance rubrics.
3. To compare learning outcomes and student perceptions between cohorts exposed to SP-based training and those undergoing traditional instruction, using OSCE performance as a key metric.
4. To explore the perceptions and experiences of key stakeholders, including leadership, SP trainers, students and SPs regarding the SP programme.

## Methods

### Medical programme details

The medical programme at the host institution is a six-year programme in which the first year is dedicated to basic science and foundational courses, while years 2, 3 and 4 follow a problem-based learning (PBL) curriculum through an integrative approach based on various body systems. Years 5 and 6 focus mainly on clinical training. Students are divided into small groups and complete rotations across disciplines such as Obstetrics & Gynaecology, Paediatrics, Internal Medicine and Clinical Electives in year 5, while Surgery (including Orthopaedics, Urology, Anaesthesia, and Accident & Emergency), Family Medicine, Psychiatry, ENT and Ophthalmology rotations are scheduled in year 6 [20].

The undergraduate medical programme at the host institution is based on a framework that consists of seven domains, each comprising a set of pre-defined competencies, programme learning outcomes, course learning outcomes and specific/session learning outcomes. The clinical competencies are assessed during clerkship rotations mainly through continuous workplace-based assessment by faculty members, student portfolios, structured mini-CEX and objective structured clinical examination (OSCE) [21].

### Study design

This study employed an explanatory sequential mixed-methods design, combining a quantitative quasi-experimental and correlational approach with a qualitative exploratory phase. It was conducted at a medical school in the Kingdom of Bahrain between January 2022 and June 2023.

The study was structured in two sequential phases:

- Phase 1 (Quantitative): focused on the development, training and evaluation of SPs, as well as their effectiveness in teaching OB-GYN history-taking skills.
- Phase 2 (Qualitative): explored stakeholder perceptions on SP implementation through in-depth interviews, building on quantitative findings.

#### Quantitative phase

The quantitative phase comprised two main parts:

- Part A: Recruitment, training and validation of SPs.
- Part B: Evaluation of the effectiveness of the SP training among fifth-year undergraduate medical students during their OB-GYN clerkship rotation.

Part A involved training selected SP candidates, and of the five candidates initially recruited, two women completed the comprehensive SP training programme and participated in the evaluation.

After the SPs were successfully trained, Part B of the study evaluated the effectiveness of the SP training. This was conducted among fifth-year undergraduate medical students during their OB-GYN clerkship rotation. Of the total sample of 104 students, 98 signed the informed consent and participated in the study. The effectiveness of the SP programme for delivering competency in history-taking during the OB-GYN clerkship was rated by the SPs, students and faculty, respectively. The details of the training session, the scenarios and all the evaluation forms are provided as supplementary files.

### Procedures

To address cultural and religious sensitivities throughout the SP training programme—both from SP and student perspectives—the scripts were written with emotional cues that were to be displayed for sensitive questions (e.g., embarrassment, hesitation). Students were trained to use a respectful and empathetic tone with careful phrasing of questions. Based on the sensitivity of the script, several role-play sessions were conducted with SPs, trainers, clinical faculty and student volunteers. During briefing and debriefing, issues related to religious and cultural norms were constantly emphasised. SPs were also encouraged to reflect upon the sessions with students and provide insights into whether they felt respectful/well understood or intimidated/uncomfortable [8–10, 22, 23]. Examples of sensitive terms and teaching strategies are shown in Table 1.

**Table 1:** Sensitive terms & teaching strategies employed to address cultural and religious sensitivity

Examples of some sensitive terms	Student training strategy used in our program*
Sexual Intercourse/Coitus	Do you get intimate with your husband? How is your relationship with your husband inside the bedroom?
Post-coital Bleeding	Have you had any bleeding after being intimate with your husband?
Menstruation	When was your last monthly cycle?
Vaginal discharge	Have you noticed any unusual discharge from your private parts?
Dyspareunia	Do you feel any pain or discomfort when you get intimate with your husband or during marital relations?
Contraception	Have you used any method to avoid or delay pregnancy?
Pregnancy loss	Have you had any pregnancies that ended before reaching full term?
History of elective termination of pregnancy	Have you had any pregnancies that you had to end for any medical or personal reasons?
Smoking	Do you use any form of tobacco?
Alcohol	Do you consume any beverages that contain alcohol?

*These strategies are based on expert consensus among trainers/SPs and clinical faculty, literature review and ongoing practices in regional hospitals and medical institutions. The strategy might vary based on the context of each scenario, and SPs are also trained to answer accordingly

#### Scenario and checklist development

Two clinical scenarios related to OB-GYN, ‘pre-eclampsia’ and ‘early pregnancy bleeding’, were meticulously developed. Structured assessment checklists and evaluation rubrics were created for each scenario to standardise the evaluations.

#### Validity assessment

Content validity of the scenarios and checklists was confirmed by one internal and two external medical experts. Face validity was assessed by five fourth-year medical students, two specialists in medical education and two language experts to ensure clarity, accuracy and ease of understanding.

### Recruitment and training of SP trainees

#### Recruitment and training of SPs

Potential SPs were recruited through a purposive sampling strategy. Announcements were circulated via email, noticeboards and by word of mouth among staff within the university and affiliated hospital networks, inviting applications from individuals with good communication skills, the ability to portray symptomatic emotional and/or behavioural aspects, the ability to give constructive feedback and the willingness to give consent for photograph/video. Interested candidates completed an initial screening to ensure they met the selection criteria. The research team assessed their availability, language proficiency, comfort with role-playing sensitive topics and alignment with the objectives of the SP programme. Preference was given to individuals with prior acting experience or a healthcare background, although this was not mandatory. Of the five candidates initially selected, two female SPs completed the structured training and participated in the study.

#### SP training program

The design of the SP training programme was informed by an extensive review of literature related to SP use in OB-GYN, with a special focus on communication skills and history-taking in undergraduate medical education. We reviewed peer-reviewed articles, scoping reviews and best practice guidelines published between 2011 and 2021 (10 years prior to the start of data collection), across databases such as PubMed, Scopus and MedEdPORTAL. Search terms included *‘simulated patients*,*’ ‘OB-GYN history-taking*,*’ ‘communication skills*,*’ ‘SP training strategies*, and *‘cultural sensitivity in medical education.’* This review helped identify key components such as emotional realism, standardisation techniques, communication phrasing for sensitive topics and feedback mechanisms.

Expert consensus on the training content and delivery strategies was established using a modified nominal group technique. The panel included two senior OB-GYN faculty with more than 10 years of teaching experience, two SP programme coordinators/trainers and two medical education experts. The team participated in three semi-structured consensus meetings, where proposed training strategies, culturally sensitive scripts, recommended vocabulary and response cues were reviewed and refined. Decisions were finalised through open discussion and majority agreement, rather than formal Delphi rounds. This collaborative process ensured alignment with both educational goals and regional cultural values.

The training lasted for 2 days with an interval of one week in between. It was structured into two detailed sessions:

- Session 1 focused on memory retention, attitude, professionalism, confidence and communication skills.
- Session 2 emphasised emotional realism, adaptability in unexpected situations and constructive feedback delivery.

The effectiveness of the training was systematically assessed using a Likert scale ranging from ‘Very Poor’ to ‘Very Good.’ The session checklists and feedback forms are provided as supplementary files.

#### Pilot testing

SPs underwent supervised practice sessions, including:

- Role play supervised by faculty
- Peer-to-peer refinement sessions
- Structured feedback loops to enhance role authenticity and response consistency

#### Evaluation of SP training

The effectiveness of the SP training was independently assessed by four expert raters who were licensed obstetricians and gynaecologists with no prior involvement in the research team. They evaluated the performance of the SPs and students using structured observational checklists, covering.

- Student performance across both clinical scenarios during four clerkship rotations
- SP performance in each scenario as judged by faculty raters
- Student assessment of SP performances
- SP self-assessments and their evaluations of student behaviours, including individualised feedback on student performance and areas for improvement

#### Validation procedures

SP portrayals were independently assessed by four clinical faculty experts (raters) using a validated observational rating scale.

Detailed scenario-specific observational checklists and rubrics were employed to evaluate the authenticity of SPs in portraying patient roles. The checklist items included specific clinical presentations as outlined in scenarios related to pre-eclampsia and early pregnancy bleeding. Performance in these scenarios was rated using a five-point Likert scale ranging from ‘missed’ (0) to ‘outstanding’ (4).

To evaluate the effectiveness of the SP training model, a quasi-experimental study was also conducted as a continuation of the SP training. The study compared the performance of two groups of students in the final OSCE examination, with a sample size of 90 in each group:

- Experimental Group: Students who underwent SP training as part of their OB-GYN history-taking preparation.
- Control Group: Students who did not undergo SP training.

### Statistical analysis

Data analyses were performed using SPSS software (version 26) and R software (version 4.2.0). The inter-rater reliability among faculty and SP evaluators was determined using Fleiss’ Kappa, Cohen’s kappa and intraclass correlation coefficients (ICC). Reliability was measured via test-retest comparisons across scenario portrayals by each SP. Independent t-tests compared mean OSCE scores between the experimental and control groups to ascertain the impact of SP training on student competency in history-taking, and statistical significance was established at a 95% confidence interval.

#### Qualitative phase

In-depth interviews using a semi-structured guide designed specifically for each group were conducted in this phase.

### Participants

The purposive sampling method was followed in this study because it enabled the exploration of diverse views on advantages, challenges and management strategies among four groups of stakeholders. A formal open email was sent to potential participants, describing the study objectives, methodology, inclusion criteria, possible risks, confidentiality and their right to withdraw from the study. However, there was no prior contact made with the participants regarding the study. Four different population samples were recruited for wider perspectives, and selection was based on the following criteria:

- Leadership/administration (to share insights on institutional goals, infrastructure, cost of training, policy/procedures on SP recruitment, etc.): directors of simulation centres, heads of SP programmes and chairpersons of clinical departments (*n* = 4) with ≥ 3 years of experience leading SP programmes in a medical school.
- Trainers/facilitators (nine faculty trainers to provide their views on SP training methodologies, retention rate, curriculum integration and delivery, etc.): those with a minimum of 2 years of experience in training SPs in this institution (*n* = 10), with ≥ 3 years of experience in training SPs in a medical school.
- Fifth-year students (to share their end-user experiences and learning difficulties): those who are taught/assessed using SPs for ≥ 3 years (*n* = 7).
- Simulated patients (to assess the difficulties in logistics, training modules, communication strategies, etc.): those with ≥ 3 years of experience as SPs in this institution (*n* = 4).

The final sample size was 25, and care was taken to include both genders in all groups except leadership, as the male participants held all the key positions during the data collection phase of this study. Initially, 28 participants volunteered; however, three of them did not consent to audio recording and were excluded from the study.

Collecting data from several sources enabled us to triangulate the findings, thereby increasing the validity and credibility of the study outcomes. Moreover, engaging all stakeholders in focused research helps promote inclusivity and provides a non-threatening opportunity for all to voice their views.

### Interview guide

The interview guides were developed based on an extensive review of the literature on the use of simulated patients (SPs) in healthcare and medical education. Key sources included studies on the effectiveness of SP training, role portrayal, standardisation, communication skill development, assessment reliability and stakeholder perspectives in SP-based programmes [22, 24–39]. Particular attention was given to research conducted in traditionally conservative contexts such as the Middle East. These studies explore gender norms and cultural appropriateness in detail, along with SP implementation [8–11, 40–46]. The review informed the design of group-specific questions that aligned with the study objectives. The content validity and construct validity were checked by three independent education experts and two qualitative analysis experts. Before implementation, the guide was also pretested with a small group of five participants (sixth-year students) to assess the clarity of questions and challenges in interpretation. The feedback received from the experts and students was applied to ensure the effectiveness of the guide.

### Data collection

Data were collected through in-depth interviews that were conducted both face-to-face and via Zoom technology, based on individual preferences and convenience, by APK and AAA, who have > 5 years of experience in qualitative research. To ensure adequate privacy, only the researcher and the participant were present during the interviews, which each lasted 30 to 45 min. Written/digital informed consent was obtained from each participant before the start of the study after thoroughly explaining all aspects of the study, including audio recording, rights to withdraw and confidentiality.

The participants were addressed using codes assigned to them to maintain confidentiality. All interviews were audio-recorded and stored in a dedicated password-protected folder shared only among the core team involved in the qualitative analysis. The questions were open-ended and presented to the participants using a neutral tone to avoid bias and generate a true response. The interviews were stopped when data saturation was reached. No follow-up interviews were conducted, nor were any field notes taken. Also, no transcripts were reverted to the study participants for further corrections. The analysis depended only on the audio recording and the verbatim transcripts.

### Data analysis

Braun and Clarke’s [47] approach to thematic analysis was employed in this study to guide the process of qualitative data analysis. All audio recordings of interviews were thoroughly transcribed in English by two authors who originally conducted the interviews. Another expert assessed the transcripts for accuracy and clarity, followed by consensus-building discussions to finalise the thematic framework. The participants’ identifying information was omitted, and the conversations were assigned based on their designation/role (e.g., student, leader, trainer, or SP). All of the transcripts were subsequently imported into the MAXQDA software (24 Analytic Pro trial version) for data sorting, and inductive methods were used to generate codes and themes. The first step involved thoroughly reading the transcripts multiple times to become immersed in the data. The meaning units of data were then extracted line by line. Each meaning unit was simplified into codes that captured the unique experiences of individuals. Subsequently, all codes were rigorously organised and merged into potential sub-themes and overarching themes.

The qualitative component of this study was led by researchers with backgrounds in medical education and clinical practice. Both authors who conducted the interviews had > 5 years of experience in qualitative research and had previously worked with the SP programme in administrative and teaching capacities. While this insider perspective allowed for a nuanced understanding of the institutional context, we acknowledged the risk of bias in the data interpretation. To mitigate this, all transcripts were coded independently by multiple team members, and peer-debriefing was conducted to refine emerging themes. We maintained reflexive journals during the analysis phase to document decisions and reflect on how our roles and assumptions may have influenced the interpretation.

### Ethical considerations

Ethical approval (Approval No. E013-PI-11/19) and funding support (Grant No. G07/AGU-11/19) were obtained from the institutional ethics committee. Furthermore, written informed consent was obtained from all participants, and confidentiality and voluntary participation were strictly maintained throughout the study.

## Results

### Quantitative phase

**Fig. 1:**
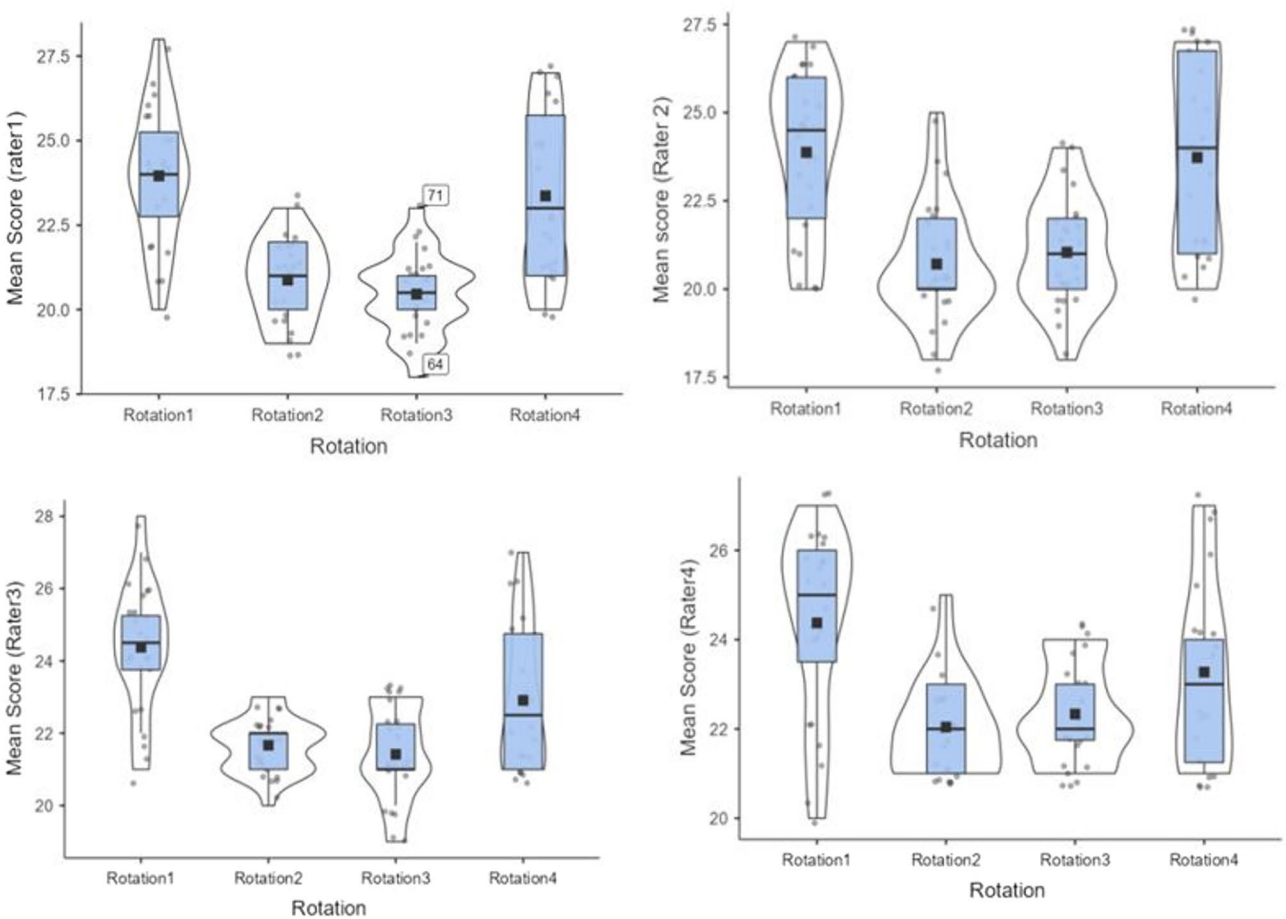
Box plots showing mean scores given by raters across all clinical rotations

For validation purposes in this phase of the study, four students participated in a mock session to test the case scenarios and simulated patient (SP) performance. However, the data were not included in the final analysis. The main study involved 94 students (23 males, 71 females) who conducted history-taking sessions with the SPs. The box plots (Fig. 1) show that the mean scores assigned by raters across all rotations display a similar pattern. The mean scores given by all raters in rotations 2 and 3 were lower when compared to those in rotations 1 and 4.

**Table 2:** Inter-Rater reliability across scenarios

Scenario	Domain/Scale	Reliability Measure (Kappa)	Internal Consistency (Cronbach’s Alpha)
Scenario 1	Overall scale	0.31	0.95
	Presenting complaints	0.63	
	Current obstetric history	0.55	
	Past obstetric history	0.61	
	Gynaecological history	0.61	
	Medical/surgical history	0.62	
	Family/social history	0.66	
	Professionalism & communication	0.22	
Scenario 2	Overall scale	0.22	0.92
	Presenting Complaints	0.52	
	Rule Out Differential Diagnosis	0.62	
	Obstetric History	0.39	
	Gynaecological History	0.55	
	Medical/Surgical History	0.64	
	Family/Social History	0.58	
	Professionalism & Communication	0.25	

For each scenario, faculty raters evaluated the performance of SPs, and the mean scores, standard deviation and Cronbach’s alpha were calculated (Table 2). The inter-rater reliability for different subscales in Scenario 1 was assessed using Fleiss’ Kappa, which measures agreement among multiple raters (Table 2). The kappa values ranged from 0.22 to 0.66, indicating moderate to substantial agreement across most subscales. The highest agreement (kappa = 0.66) was observed for Family/Social History, suggesting strong consistency in ratings for this category. Professionalism & Communication had the lowest agreement (kappa = 0.22), indicating higher variability among raters in assessing communication skills and professional demeanour. For Scenario 2, inter-rater agreement showed a similar trend, with kappa values ranging from 0.25 to 0.64 (Table 2). Overall, the results indicate moderate to substantial agreement among raters across most subscales in both scenarios, with slightly lower consistency in Scenario 2 compared to that in Scenario 1.

Fleiss’ kappa values ranged from moderate to substantial across various subscales, suggesting that raters consistently identified key history components due to well-defined evaluation criteria or clearer benchmarks, except for Professionalism & Communication. The competencies in this domain are more susceptible to subjective interpretation and underscore the necessity for clearer behavioural anchors.

The intraclass correlation coefficient (ICC) was used to measure the consistency of ratings among different raters. The results indicate moderate agreement in most evaluations (Table 3). For instance, evaluation of students by raters showed ICC values of 0.469 (Scenario 1) and 0.34 (Scenario 2), indicating moderate to fair agreement, with Scenario 2 showing slightly lower consistency. Evaluation of SPs by raters demonstrated higher reliability, with SP 1 (ICC = 0.588) and SP 2 (ICC = 0.594), suggesting good consistency in SP assessments compared to that in student evaluations. The lower ICC for student evaluations suggests greater variability in how students were rated across different scenarios, while the higher ICC for SP evaluations indicates better agreement among raters in assessing the performance of SPs.

**Table 3:** Inter-rater reliability

	Intra-Class Coefficient (ICC)
Evaluation of students by Raters (Scenario 1)	0.469
Evaluation of students by Raters (Scenario 2)	0.34
Evaluation of SP* 1 by Raters	0.588
Evaluation of SP* 2 by Raters	0.594

*Simulated Patient

Test–retest reliability was measured for each rater (R1 to R4) across four rotations to assess the consistency of ratings over time (Table 4). The highest reliability was observed in Rotation 1, where all raters showed strong agreement, indicating excellent stability in ratings. In Rotations 2 and 3, reliability remained high but showed slight reductions across raters, particularly in R3 and R1. Rotation 4 showed the lowest reliability, with scores ranging from 0.47 (R1) to 0.65 (R3), indicating greater fluctuations in ratings over time.

**Table 4:** Test–retest reliability

	R*1	R*2	R*3	R*4
Rotation 1 (Scenario 1/Scenario 2)	0.81	0.92	0.97	0.98
Rotation 2 (Scenario 1/Scenario 2)	0.74	0.77	0.60	0.79
Rotation 3 (Scenario 1/Scenario 2)	0.70	0.76	0.79	0.87
Rotation 4 (Scenario 1/Scenario 2)	0.47	0.60	0.65	0.58

*R- Rater

To assess the effect of SP training on students and compare their performances in the final OSCE, an independent samples t-test was conducted to compare the mean values between the control and intervention groups (Table 5). The results indicated no statistically significant difference between the two groups [t(178) = −1.35, *p* = 0.179 (two-tailed)]. The effect size, measured using Cohen’s d, was d = −0.201, indicating a small effect. Nevertheless, given that the p-value exceeded the conventional alpha level of 0.05, we failed to reject the null hypothesis, suggesting that the intervention did not produce a meaningful impact compared to the control condition.

**Table 5:** OSCE evaluation

	*N*	Mean ± SD	T statistic	*P* value	Cohen’s d
Control Group	90	64.51 ± 12.57	−1.348	0.179	−0.201
Intervention Group	90	66.84 ± 10.52			

The analysis of evaluation outcomes demonstrated clear distinctions between standardised patients (SP1 and SP2) and student performances. Faculty evaluation of SP1 revealed high ratings for maintaining standardisation (up to 92%) and fitting into the role (85%), although there was notable variability in observing the scenario (as low as 26%), reflecting differences in rater perception across specific competencies. In contrast, SP2 received more consistent and higher ratings across domains, particularly in communication with students (96%) and standardisation (98%), with relatively lower but still moderate scores in completing the history and observation tasks.

When evaluating students, SP1 highlighted strong performance in clarity of communication (88%) and addressing concerns (93%), while SP2 echoed similar trends, commending the students’ ability to clarify information (86%) and show empathy (85%). However, the ratings for encouraging questions and expressing empathy were slightly lower. Student evaluations of the overall experience of SPs were highly positive, with the strongest agreement being with the professionalism, communication skills and value of SPs in training (up to 90%). Nevertheless, slightly lower comfort levels were reported during interactions (69%), suggesting room for improvement in student–SP rapport.

**Fig. 2:**
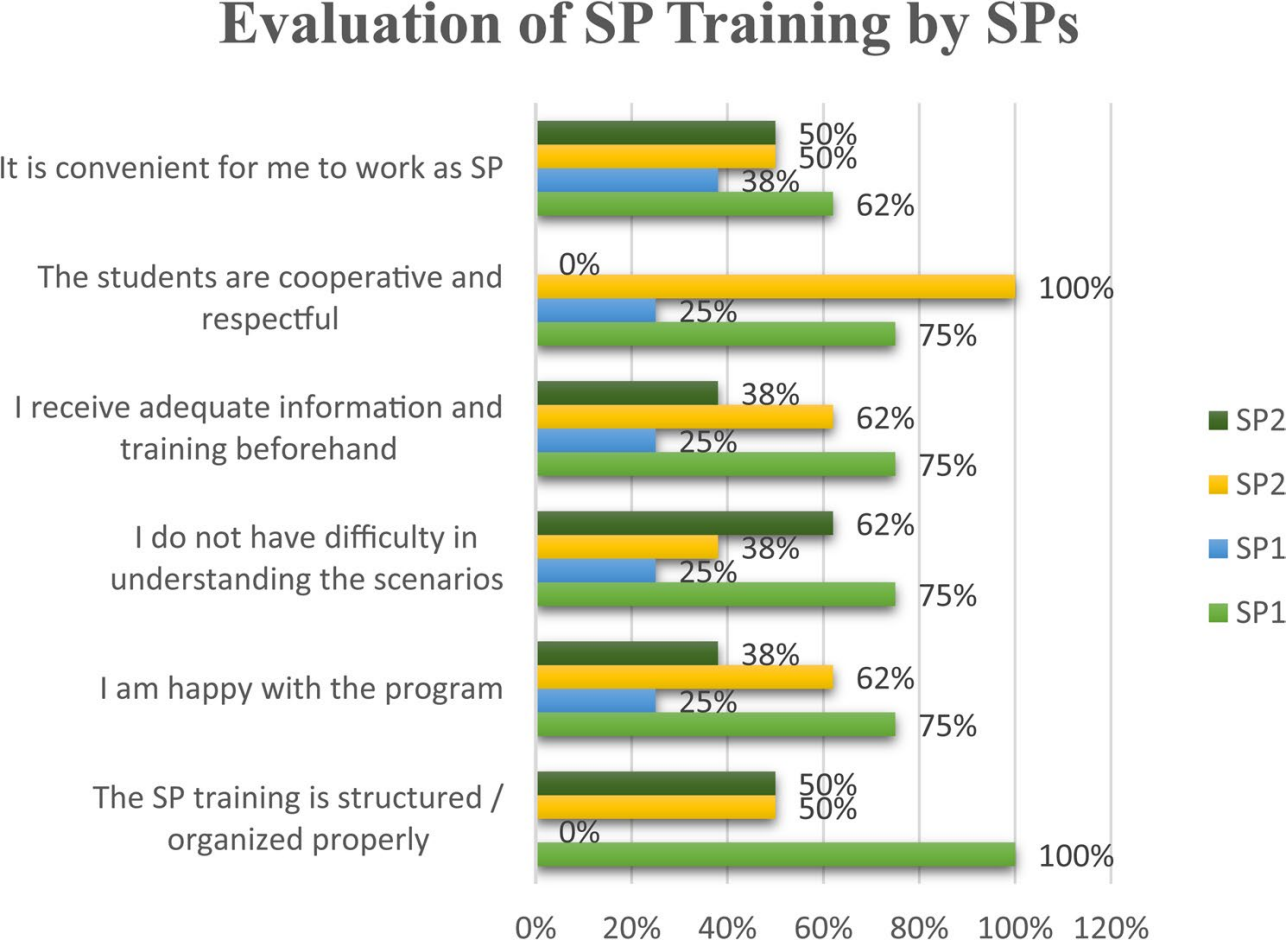
Evaluation of SP training by SPs

The evaluations of SP training by the SPs themselves underscored the effectiveness of the training sessions with high approval ratings while also highlighting areas of potential improvement to further enhance SP readiness and performance reliability (Fig. 2).

### Qualitative phase

We identified three overarching themes that emerged clearly from participants’ narratives:

1. The simulated patient (SP) programme as a comparable educational strategy for clinical competencies development,
2. Challenges associated with the SP programme and.
3. Suggestions for improving the SP programme.

Each theme and associated sub-theme are detailed below with representative quotes.

A word cloud was generated from the codes assigned during thematic analysis using MAXQDA.

### SP programme as a comparable educational strategy for clinical competencies development

#### Address ethical and social challenges associated with practising on real patients

Participants described numerous barriers to working effectively with real patients during clinical postings, often citing limited opportunities for hands-on learning. One leader noted, *‘Many patients don’t want to be disturbed*,* and maintain their confidentiality. With growing awareness of patient rights and ethics*,* it is becoming difficult for our students to practice clinical skills on real patients’* (Leader 3). Students reflected on how shorter hospital stays and improved healthcare delivery meant fewer inpatients to engage, leading to reduced clinical exposure. Beyond logistical constraints, participants also emphasised the emotional and cultural complexities of patient interactions: *‘Some patients may not talk to the opposite gender freely*,* especially in history related to mental health*,* sexual health or gender-related concerns*,*’* explained another faculty member (Leader 2). These limitations were compounded by the unpredictability of patient responses, which, according to students, often added to their cognitive load. One student shared, *‘When we usually go to take history from real patients*,* they sometimes respond to something which is irrelevant to their case. That affects our history-taking and the provisional diagnosis’* (Student 3). These experiences left students feeling uncertain and unfocused, struggling to extract clinically relevant information.

#### Provide a safe and structured space for learning

Participants consistently described SP training as a safe yet realistic environment that supports repeated practice without the risk of harming actual patients. One student reflected,

‘They let us practise things like clinical skills and having sensitive or tough conversations without actually… worrying about hurting anyone, or fear of getting judged by others. It’s kind of like building confidence in a safe space,’ (Student 6).

This sentiment was echoed across all participants, including trainers, SPs and faculty. They all emphasised that the risk-free nature of simulation contributed to reduced anxiety and a stronger foundation in clinical competence. Beyond technical proficiency, the SP programme is also seen as instrumental in cultivating softer skills such as empathy, professionalism and rapport building. All participants agreed that it is harder to teach through lectures or passive observation.

‘SPs can be used for very difficult conversations such as breaking bad news, sexual history, menstrual history, cancer history and HIV history, which most students might find difficult to discuss in the real world,’ (SP4).

#### Opportunity for standardised and equal exposure to multiple health conditions

Participants highlighted the unique value of the SP programme in offering standardised exposure to a broad spectrum of clinical scenarios. Unlike real patients, whose conditions and availability are unpredictable, SPs allow for the consistent delivery of diverse medical cases. One simulated patient reflected on this advantage:

‘They get exposed to different types of cases and personalities. Do you think you will get so many cases of Type 2 diabetes with an infected wound on the same day for all the students? No, it’s not possible. Similarly, a case of mania or a case of depression. So, it’s really important that the college takes more steps,’ (SP2).

Faculty and trainers echoed this perspective, recognising how scripted SP scenarios not only standardise training but also ensure fairness across student cohorts. SPs can be coached to portray rare, complex or sensitive cases that students may not otherwise encounter.

‘All students get the same opportunity, and I think it makes their exam more standardised. It also helps us to create rare cases, difficult scenarios, which the student may see in the future—cases that are not available in this country but may be seen in other countries,’ *(*SP3).

### Challenges associated with the SP programme

#### Lack of realism and authenticity

Participants frequently reflected on how the perceived authenticity of SP interactions was shaped by familiarity and performance quality. Several SPs shared that students, having seen them repeatedly, often struggled to fully suspend disbelief. *‘Because they’ve seen us here many times and they know we are actors*,* not real patients*,* some of them don’t take us seriously*,*’* (SP1). Although beneficial for rapport, this familiarity can sometimes dilute the realism of the interaction and lead to reduced student engagement. Students expressed this concern, noting that even well-scripted scenarios could feel artificial. *‘Like*,* first*,* sometimes it doesn’t feel totally real*,* you know? No matter how well they act*,* there’s still that thought in the back of your mind that this isn’t a real patient. So*,* the experience isn’t always 100% realistic’* (Student 6). These limitations affect students’ ability to immerse themselves in the scenario, making it harder to respond authentically or to adapt to complex clinical situations. Trainers also pointed out another challenge: while SPs with nursing or medical backgrounds often better understood clinical nuances, they sometimes lacked the range of emotional expression needed to mimic real patient diversity. As one trainer noted, *‘We had a few nurses acting as SPs. They already have a medical background; at the same time*,* it might not be exactly the same as you see in real patients in a hospital or a health centre*,*’* (Trainer 6).

Across the board, participants acknowledged that while SPs play a critical role in clinical education, they cannot fully replicate the unpredictability, emotional depth or physiological complexity of real patient encounters.

#### Challenges in recruitment and retention of SPs

Programme leaders described persistent difficulties in recruiting and retaining SPs, pointing to a combination of cultural, logistical and financial factors. For instance, gender norms were identified as a significant barrier, particularly for female applicants who were reluctant to participate in sessions involving examination by male students. Furthermore, language differences contributed another layer of complexity. Trainers noted that regional variations in dialects made it challenging to teach SPs how to use medically appropriate terminology.

‘I think in our culture—especially in the Gulf Cooperation Council (GCC) like Bahrain—people cannot understand the concept of SP. They are afraid or reluctant to act, especially with male students. Many people don’t understand why it is relevant and beneficial to health care.’ (Trainer 1).

In addition to these cultural hesitations, the limited awareness of the broader community of the role and value of SPs in healthcare education further discouraged participation.

Retention of SPs posed its own set of challenges. SPs frequently balanced other jobs and responsibilities, making it difficult to accommodate irregular or last-minute scheduling for training and assessments. *‘We are expected to stay here for 6 hours or so during exams. All of us work elsewhere as part-timer; so*,* we have to plan our schedules accordingly.’* (SP1). Financial compensation emerged as another concern, with several SPs expressing that the time and effort involved did not match the remuneration. *‘I think money also plays a part. Many SPs feel the fees we’re getting are not sufficient; so*,* they don’t really want to get involved in the programme. Also*,* it is tiring for SPs to repeat the same scenario multiple times.’* (SP3).

#### Challenges associated with programme designing, standardisation and implementation

Challenges in recruiting and retaining SPs had clear downstream effects on how the SP programme was designed and delivered. For instance, trainers and leaders frequently found themselves adjusting training schedules and formats based on SP availability.

‘Our schedule has to align with their schedule. They usually prefer to come in the afternoon or towards the end of the day. But our clinical training starts in the morning. So, we have to adjust that schedule.’ (Leader 4).

Unplanned absenteeism was another recurring issue that often disrupted the flow of training and exams. *‘Absenteeism is one problem. For example*,* we arranged an exam for today. Some SPs all of a sudden in the morning will say*,* ‘today I can’t come*,*’* said one trainer, describing the scramble that follows. *‘The trainers get tense. They run here and there; the entire session gets disrupted*,* even delaying some of the exams by 15 minutes… half an hour.’* (Trainer 10).

Time constraints and script complexity further compounded the difficulty encountered in effectively preparing SPs. Many felt rushed during training, struggling to inhabit roles that required emotional nuance. *‘The time given for training is very short—only a few minutes; so*,* it really puts lots of pressure on us to fully prepare for certain roles. It affects the quality of the training we provide’* (SP 2). Trainers also noted that some SPs—especially those unfamiliar with healthcare settings or lacking acting experience—had difficulty portraying the emotional states embedded in the script. ‘*They don’t have experience to act based on the script… so*,* when they’re supposed to act as if they have real pain*,* they act very normal—even for a painful script’* (Trainer 9). Several participants described the pressure to memorise long scripts that impacted the ability of SPs to remain consistent, often leading to flat or unrealistic performances.

Another challenge with the SP programme is that of confidentiality issues during assessments. Trainers reported that they could not brief SPs too far in advance about their roles in exams to preserve fairness. Moreover, the SPs occasionally found themselves caught between professional boundaries and personal empathy.

‘Even though we shouldn’t discuss these things with students, some of my colleagues feel like we have to help them. Our children also go to college. We know how they struggle. They try to help by revealing the diagnosis or giving more hints during the exam.’ (SP 2).

These well-intentioned breaches can threaten the integrity of the evaluation process and highlight the blurred lines SPs sometimes navigate between their scripted roles and real-world identities.

### Suggestions for improving the SP programme

#### Improving SP working conditions

The study participants emphasised that improving the working conditions for SPs would significantly strengthen the SP programme. Financial compensation emerged as a primary concern for SPs, and many suggested that better pay could directly influence their motivation and long-term commitment. *‘If we increase the amount*,* I think we can get more commitment and motivation from SPs.’* (Trainer 8).

Fatigue during lengthy sessions was another issue that surfaced frequently, particularly during exam days. SPs spoke of physical and mental exhaustion after sitting through back-to-back encounters without rest. *‘Scheduling breaks between the sessions is very important. Unfortunately*,* during exams*,* we sit for a long time*,* and it leads to burnout. It would be nice if they could give us a break after every six or seven students*,*’* (SP 3), acknowledging that while this change may be logistically difficult, it would significantly improve their experience.

Beyond logistics and compensation, the quality of training itself was identified as an area for improvement. Trainers highlighted the importance of providing clear, structured case guidelines and briefings to SPs on key clinical cues to maintain consistency across sessions. *‘All trainers should be given a clear guide… what information should be conveyed to the SP*,* what are the red flags*,* and what the SP should or should not say’* (Trainer 3). Participants believed that more deliberate faculty engagement in SP training could enhance the fidelity of role play and contribute to more effective learning outcomes for students.

#### Enhancing programme accessibility and flexibility

Participants highlighted the need for improved communication and scheduling structures to make the SP programme more accessible and flexible. Many pointed out that last-minute notifications often led to confusion or absenteeism, suggesting that advance scheduling and centralised communication could enhance coordination. One trainer emphasised the value of having a shared space for updates: *‘We should have somebody as backup. Also*,* I think we should have a common WhatsApp group to contact them because personal messaging is difficult. We need a separate forum for SP coordination and to disseminate some common information’* (Trainer 10). Participants felt that having such a system would streamline communication and improve accountability among SPs.

In addition to better scheduling, trainers also proposed that incorporating hybrid formats into the training process could be helpful. They felt that offering both in-person and online options could accommodate SPs with other commitments without compromising the quality of preparation. *‘Some sessions can be online… some face-to-face. We should prepare our SPs for those scenarios—for at least two days*,*’* (Trainer 8), underlining the need for regular, structured orientation regardless of the format. To further minimise disruptions, several participants recommended maintaining a roster for backup SPs who could step in when needed, to ensure that sessions could proceed smoothly even in cases of last-minute cancellations.

#### Expanding and strengthening training

The study participants reflected that to maintain the quality of the programme, it is important to have regular, structured training sessions for both SPs and students, as ongoing engagement rather than last-minute preparation is key. One student reflected on this need for continuity, saying:

‘I think regular, spaced training sessions would help a lot. Instead of just cramming for exams or focusing only on simulation centres, we could have ongoing sessions throughout the year to keep practising—not just professional skills but communication and empathy, also. It’d feel more natural and like real patient interactions’ (Student 6).

This sentiment was shared among the groups, with many suggesting that consistency would not only enhance student learning but also allow SPs to refine their acting, deepen their understanding of roles, and maintain emotional accuracy across cases.

Several participants also called for stronger clinical involvement in SP training. Trainers and leaders noted that while current facilitators were effective in guiding logistics and delivery, the lack of clinical expertise sometimes limited the depth of training, particularly when dealing with complex medical content.

‘We strongly believe having a clinical faculty during the training and validation of the SP adds more value to the team as well, because even though our trainers are good, they are not clinicians. They do not know the nuances while training our SPs’ (Leader 3). Another trainer added, ‘I recommend that some clinicians or faculty who write the script should help us in the training, especially if it contains difficult terms’ (Trainer 7).

Beyond the training structure, participants also highlighted the need for more selective and strategic SP recruitment. Many believed that the authenticity of the program could be significantly enhanced by choosing individuals with prior acting experience and a background in healthcare or familiarity with medical conditions. ‘*Colleges can recruit SPs from nurses or supporting staff who are close to healthcare*,* or it will be better if we get an SP with an acting background like me’* (SP 4). Leaders supported this idea that careful selection, especially of bilingual, motivated candidates who might themselves have a chronic illness, could add realism to the role-play and increase the educational impact for students. *‘We will try to get more educated*,* bilingual candidates who are motivated and can act*,* and if they have some disease entity that can be used*,* such as hypertension or diabetes’* (Leader 1). In parallel, there was consensus that faculty members responsible for writing scripts should also be trained to develop clear, emotionally grounded narratives. As one leader explained, *‘We must train our clinical faculty on how to simplify the scenario*,* know what they expect*,* what emotion should be displayed and what the SP’s body language should be. It should all be clearly written in the script’* (Leader 3).

## Discussion

### Validating the training of simulated patients for teaching and assessing history-taking skills of medical students

This study contributes to the growing body of literature on simulation-based education by validating a culturally responsive simulated patient (SP) training programme tailored for obstetrics and gynaecology (OB-GYN) education in the GCC region. Unlike many SP studies conducted in Western contexts, our findings shed light on the unique interplay among culture, gender norms and learner experience in conservative societies, an area still underexplored in simulation literature [8–11, 15]. Cronbach’s alpha values were found to be notably high (0.95 for Scenario 1 and 0.92 for Scenario 2), indicating excellent internal consistency of the assessment. These findings align well with existing literature, confirming the reliability of SP-based methods for assessing clinical competencies of students [24, 25].

This study importantly affirms that SP programmes offer more than just standardised assessments [26]; they serve as safe spaces for students to practice culturally sensitive communication. For instance, in conservative OB-GYN contexts, where real-patient exposure is often limited for male students, SPs offer a rare opportunity for navigating sensitive topics (e.g., menstrual health, sexual history) without fear of cultural offence or patient discomfort. This finding aligns with previous research advocating for simulation in teaching reproductive health communication, particularly in settings where modesty and gender dynamics influence patient–provider interactions [8–10, 22].

The variability in inter-rater reliability in the domain of professionalism and communication highlights a common challenge in SP assessment. There may be subjectivity in evaluating interpersonal skills [27, 28], underlining the need for more specific behavioural anchors and regular rater calibration to maintain consistency. The gradual decline in test–retest reliability across the four rotations may reflect rater fatigue or changes in student–SP dynamics [29]. Hence, periodic rater recalibration, structured breaks and refresher training may be some ways by which consistent scoring can be maintained across prolonged assessment periods.

The objective structured clinical examination (OSCE) scores between control and intervention groups revealed no statistically significant difference (*p* = 0.179, Cohen’s d = −0.201). This absence of significant differences can be attributed to various factors, including assessment design, measurement sensitivity and the nature of the skills being evaluated [30–33]. The small effect size suggests minimal practical differences between groups, implying that while SP-based training has demonstrable theoretical advantages, additional refinement in implementation strategies or duration may be necessary to yield measurable impacts on student outcomes. Interestingly, this lack of significance contrasts with the positive qualitative perceptions reported by students, suggesting a possible misalignment between what is measured and what is valued in student–SP encounters.

### Stakeholder perceptions regarding the effectiveness and execution of the simulated patient (SP) programme

#### Authenticity and engagement: limits to experiential depth

Despite the pedagogical strengths of the SP programme, stakeholders indicated a perceived deficiency in the authenticity of the SPs. In the current study, familiarity with SPs, deficient acting proficiency and an inability to convey emotional realism were identified as impediments to the SP programme. This concern has been raised in past works. When SPs do not convincingly mimic true behaviours or responses, students tend to disengage, which significantly hampers their learning experiences [48].

To address these concerns, it may be essential to enhance the training and selection processes for SPs, to ensure that they can embody the roles with greater emotional realism. Implementing rigorous screening methods and comprehensive training programmes that focus on acting techniques could significantly elevate the quality of simulations, thereby enriching the experiential learning process for students [49]. By refining these aspects of SP programmes, educational institutions can optimise the experiential learning cycle proposed by Kolb, ultimately enhancing the preparedness of future healthcare professionals.

### Operational barriers undermining experiential learning

Recruitment and retention challenges among SPs emerged as structural barriers in the present study. These difficulties were found to be particularly exacerbated by sociocultural factors, linguistic diversity, scheduling conflicts and perceptions regarding inadequate compensation. For maintaining a dependable pool of SPs, proper coordination, appropriate compensation and institutional commitment are essential, all of which represent enduring obstacles in both Western and non-Western educational environments [50, 51]. However, challenges such as systematic quality assurance, recruitment, training and the need for research and scholarship in SP methodology remain prevalent, hence the necessity for continuous improvement and adaptation to local and national conditions [51, 52].

### Strengthening the learning cycle: suggestions for improvement

Despite the operational challenges in the SP programme, our study participants suggested practical improvement strategies that align with global best practices. Such suggestions include more defined script guidelines, scheduled breaks during sessions and consistent mock drills for both SPs and students.

Engaging clinical faculty in the development of scripts and training for SPs was identified in this study as a strategy for enhancing authenticity and ensuring alignment with actual clinical expectations, as evidenced in past works [52]. This collaborative approach may effectively bridge the divide between simulation and real-world practice. Together, these suggestions point towards the need for a more integrated, collaborative and skilfully executed OB-GYN SP programme capable of enhancing both clinical precision and emotional authenticity.

The frequency and prominence of codes such as ‘Selection,’ ‘Standardisation’ and ‘Institutional Collaboration’ in the word cloud reaffirm the central concerns of participants regarding operational continuity, quality assurance and structural support for the SP programme. Integrating these recommendations into a practical framework, such as ‘Three Pillars for Sustainable SP Programmes’ (Selection, Standardisation, Support), may guide future policy implementation and quality assurance efforts.

### Limitations of the study

Although this study provides rich, multi-stakeholder insights into the SP programme within a GCC medical college, its numerous limitations must be acknowledged. First, the study was conducted in a single institution, which may limit the transferability of findings to other cultural or institutional contexts. The small sample size—both in the preliminary validation phase and qualitative inquiry, especially for leadership and SP groups—potentially constrains the breadth of perspectives. Additionally, the potential rater and social desirability biases may have influenced participant responses.

Another limitation lies in the absence of longitudinal data. The study captured perceptions at a single point in time, without tracking changes in experiences or competencies throughout the academic programme.

Lastly, the mixed methods approach was applied sequentially, and integration of qualitative and quantitative insights was conducted post hoc. Future studies may benefit from a convergent design to enable more direct triangulation and thematic alignment.

### Directions for future research

Future research could benefit from a multi-institutional approach to capture a wider diversity of experiences, as well as from longitudinal designs to evaluate the impact of SP training on student outcomes over time. Additional research is necessary to refine the evaluation criteria for communication and professionalism, which could potentially enable the incorporation of more objective measures or technological aids (e.g., video review) into the SP programme. Moreover, incorporating video-recorded SP interactions and reflective diaries could provide richer triangulation of data and deeper insight into experiential learning dynamics. Comparative studies involving real patient interactions and hybrid simulation models may also help further elucidate the unique contributions and limitations of SPs in the medical training environment. Innovative approaches, such as the use of immersive and in situ simulations, can be explored in the region to enhance training effectiveness. While there is a clear recognition of the benefits and potential of simulation programs in the Gulf, there is a pressing need for strategic investment, infrastructure development, and adherence to international best practices to realise these benefits and align with global standards.

## Conclusion

A set of validated assessment tools for training simulated patients (SPs) for history-taking in OB-GYN was developed from this study. This training programme was found to be effective for enhancing the skills and knowledge required by SPs to effectively simulate OB-GYN patient roles. Our study highlights the critical contribution of simulated patient (SP) programmes to experiential learning and to the development of clinical competence in the Gulf Cooperation Council (GCC) medical education. Stakeholders in this study valued SPs for their ability to provide safe, structured and standardised training environments; however, they identified significant challenges, including limited authenticity, recruitment difficulties and logistical barriers. Addressing these challenges through targeted SP selection, enhanced training and institutional support can help strengthen the effectiveness of the programme. Given that healthcare education in the GCC continues to evolve, investing in sustainable, culturally adapted SP programmes will be key to cultivating a competent, empathetic and practice-ready healthcare workforce.

## Supplementary Information

Supplementary Material 1.

Supplementary Material 2.

Supplementary Material 3.

Supplementary Material 4.

Supplementary Material 5.

Supplementary Material 6.

Supplementary Material 7.

Supplementary Material 8.

Supplementary Material 9.

Supplementary Material 10.

Supplementary Material 11.

## Data Availability

No datasets were generated or analysed during the current study.

## Abbreviations

SPs: Simulated Patients
OB-GYN: Obstetrics and gynaecology
OSCE: Objective structured clinical examination
ICC: Intra-class coefficient

## References

[1] Zhang J, Cheng M, Guo N, et al. Standardized patients in teaching the communication skill of history-taking to four-year foreign medical undergraduates in the department of obstetrics and gynaecology. BMC Med Educ. 2019;19:108. 10.1186/s12909-019-1541-y. 30987621 10.1186/s12909-019-1541-y PMC6466762

[2] Patel R. Enhancing history-taking skills in medical students: a practical guide. Cureus. 2023;15(7):e41861. https://www.cureus.com/articles/167890-enhancing-history-taking-skills-in-medical-students-a-practical-guide. 37581148 10.7759/cureus.41861 PMC10423320

[3] Millstein LS, Rosenblatt P, Bellin MH, Whitney L, Eveland SR, Lee MC, et al. Advance care planning and communication skills improve after an interprofessional team simulation with standardized patients. Palliat Med Rep. 2022;3(1):123–31. 36059907 10.1089/pmr.2021.0086 PMC9438443

[4] Hagey JM, Toole J, Branford K, Reynolds T, Livingston E, Dotters-Katz SK. Understanding sexual complaints and history taking: a standardized patient case on dyspareunia for obstetrics and gynecology clerkship students. MedEdPORTAL. 2020;16(1):11001. 33150201 10.15766/mep_2374-8265.11001 PMC7597941

[5] Goh PRQ, Ng GYJ, Shorey S, Lim S. Impacts of standardized patients as a teaching tool to develop communication skills in nursing education: a mixed-studies systematic review. Clin Simul Nurs. 2023;84:101464.

[6] Baugh RF, Baugh AD. Cultural influences and the objective structured clinical examination. Int J Med Educ. 2021;12:22–4. 33507878 10.5116/ijme.5ff9.b817 PMC7883802

[7] Bonifacino E, Corbelli J. (2020). The female sex- and gender-specific history and examination. In: Tilstra, S.A., Kwolek, D., Mitchell, J.L., Dolan, B.M., Carson, M.P. (eds) Sex- and Gender-Based Women's Health. Springer, Cham. 10.1007/978-3-030-50695-7_3. Print ISBN 978-3-030-50694-0, Online ISBN978-3-030-50695-7.

[8] Aryal S, Atreya A. History taking in gynecology revisited. Acta bio-medica Atenei Parmensis. 2022;92(6):e2021554. 10.23750/abm.v92i6.11940 PMC8823587 35075073

[9] Rizk DEE, El-Zubeir MA, Al-Dhaheri AM, Al-Mansouri FR, Al-Jenaibi HS. Determinants of women’s choice of their obstetrician and gynecologist provider in the UAE. Acta Obstet Gynecol Scand. 2005;84(1):48–53. 15603567 10.1111/j.0001-6349.2005.00705.x

[10] Rizk DEE, El-Safty MM. Female pelvic floor dysfunction in the Middle East: a tale of three factors—culture, religion and socialization of health role stereotypes. Int Urogynecol J. 2006;17(5):436–8. 10.1007/s00192-005-0055-9 16411017

[11] Rashidian M, Minichiello V, Knutsen S, Ghamsary M. (2020). Western, Asian, and Middle Eastern Societies’ cultural attitudes and barriers impacting the management of Sexual Health Care. In: Rowland D, Jannini E. (eds). Cultural Differences and the Practice of Sexual Medicine. Trends Androl Sex Med. Springer, Cham. 10.1007/978-3-030-36222-5_10. Print ISBN978-3-030-36221-8, Online ISBN978-3-030-36222-5.

[12] Puddister S, Ali-Saleh O, Cohen-Dar M, Baron-Epel O. Health may be compromised by social interactions depending on culture among postpartum Arab and Jewish Israeli women. BMC Pregnancy Childbirth. 2020. 10.1186/s12884-020-03168-4. 10.1186/s12884-020-03168-4 PMC7441553 32825830

[13] Brookmeyer KA, Coor A, Kachur RE, Beltran O, Reno HE, Dittus PJ. Sexual history taking in clinical settings: a narrative review. Sex Transm Dis. 2020;48(6):393–402. 10.1097/OLQ.0000000000001319 33093285

[14] Fayed MA, Ramadan WA, Al-Omran F, Alakhtar A. Simulation training in the Middle East: Experts’ viewpoint on current status vs. future trends. J Clin Res Bioeth. 2016;7:1000287. 10.4172/2155-9627.1000287.

[15] Katoue MG, Cerda AA, García LY, Jakovljevic M. Healthcare system development in the Middle East and North Africa region: Challenges, endeavors and prospective opportunities. Front Public Health. 2022;10:1045739. 10.3389/fpubh.2022.1045739. PMID: 36620278; PMCID: PMC9815436. 10.3389/fpubh.2022.1045739 PMC9815436 36620278

[16] Khoja T, Rawaf S, Qidwai W, Rawaf D, Nanji K, Hamad A. Health care in Gulf Cooperation Council countries: a review of challenges and opportunities. Cureus. 2017;9(8). https://www.ncbi.nlm.nih.gov/pmc/articles/PMC5650259/. 10.7759/cureus.1586 PMC5650259 29062618

[17] Balla S, Mohamed I. The Gulf is lagging behind on gender equality. here’s how it can catch up [Internet]. Washington (DC): Atlantic Council; 2022 [cited 2025 Sep 2]. Available from: https://www.atlanticcouncil.org.

[18] Fahad S. Cultural aspect of sustainable development in the GCC. https://www.jcsronline.com/wp-content/uploads/2023/01/Volume6Issue11Paper1.pdf.

[19] Kolb DA. Experiential learning: [internet]xperience as [internet]he source of learning and development. Research Gate. Prentice-Hall;1984. https://www.researchgate.net/publication/235701029_Experiential_Learning_Experience_As_The_Source_Of_Learning_And_Development.

[20] خارطة المنهج. | Arabian Gulf University. Agu.edu.bh. 2024. https://www.agu.edu.bh/ar/page/khartt-almnhj. Cited 2025 May 28.

[21] أساليب التقييم. | Arabian Gulf University. Agu.edu.bh. 2024. https://www.agu.edu.bh/ar/node/168. Cited 2025 May 28.

[22] Young OM, Parviainen K. Training obstetrics and gynecology residents to be effective communicators in the era of the 80-hour workweek: a pilot study. BMC Res Notes. 2014. 10.1186/1756-0500-7-455. 10.1186/1756-0500-7-455 PMC4105231 25030271

[23] Elson M. Cultural Sensitivity in OB/GYN: The Ultimate Patient-Centered Care. MedEdPORTAL. 2009;5:1658. 10.15766/mep_2374-8265.1658.

[24] Williams B, Song JJY. Are simulated patients effective in facilitating development of clinical competence for healthcare students? A scoping review. Adv Simul. 2016. 10.1186/s41077-016-0006-1. 10.1186/s41077-016-0006-1 PMC5796606 29449975

[25] Sandeva MG, Tufkova S, Ketev K, Paskaleva D. Evaluating the effectiveness of simulation training in obstetrics and gynecology, pediatrics and emergency medicine. Folia Med (Plovdiv). 2019;61(4):605–11. 32337878 10.3897/folmed.61.e47961

[26] Haruta J, Nakajima R, Monkawa T. Development of a validated assessment tool for medical students using simulated patients: an 8-year panel survey. BMC Med Educ. 2024. 10.1186/s12909-024-05386-2. 10.1186/s12909-024-05386-2 PMC11007881 38600531

[27] Keifenheim KE, Teufel M, Ip J, Speiser N, Leehr EJ, Zipfel S, et al. Teaching history taking to medical students: a systematic review. BMC Med Educ. 2015;15:159. 10.1186/s12909-015-0443-x. 26415941 10.1186/s12909-015-0443-x PMC4587833

[28] Boucetta N, El Alaoui M. Clinical simulation training for the adequate management of obstetrics emergencies: a narrative review. Medwave. 2023. 10.5867/medwave.2023.10.2712. 10.5867/medwave.2023.10.2712 37922430

[29] Paravattil B, Wilby KJ. Optimizing assessors’ mental workload in rater-based assessment: a critical narrative review. Perspect Med Educ. 2019;8(6):339–45. 10.1007/s40037-019-00535-6. 10.1007/s40037-019-00535-6 PMC6904389 31728841

[30] Bosse HM, Schultz JH, Nickel M, Lutz T, Möltner A, Jünger J, et al. The effect of using standardized patients or peer role play on ratings of undergraduate communication training: a randomized controlled trial. Patient Educ Couns. 2012;87(3):300–6. 22137189 10.1016/j.pec.2011.10.007

[31] Qureshi AA, Zehra T. Simulated patient’s feedback to improve communication skills of clerkship students. BMC Med Educ. 2020;20(1):15. 31941466 10.1186/s12909-019-1914-2 PMC6964074

[32] Gorski S, Prokop-Dorner A, Pers M, Stalmach–Przygoda A, Malecki Ł, Cebula G, et al. The use of simulated patients is more effective than student role playing in fostering patient-centred attitudes during communication skills training: a mixed method study. BioMed Res Int. 2022. 10.1155/2022/1498692. 10.1155/2022/1498692 PMC9789908 36573197

[33] Wollney EN, Vasquez TS, Stalvey C, Close J, Markham MJ, Meyer LE, et al. Are evaluations in simulated medical encounters reliable among rater types? A comparison between standardized patient and outside observer ratings of osces. PEC Innovation. 2023;2:100125. 37214504 10.1016/j.pecinn.2023.100125 PMC10194306

[34] Babu SA. Clinical obstetrics and gynecology: history taking, discussions, and practical viva voce topics. Wolters Kluwer; 2019.

[35] van der Vleuten CPM, Swanson DB. Assessment of clinical skills with standardized patients: state of the art. TLM. 1990;2(2):58–76. 10.1080/10401334.2013.842916 24246102

[36] Ozcelik R, Ayhan H. The effect of standardized patient use in teaching preoperative care practices on students’ knowledge, skills, and anxiety in clinical practice. CSN. 2021;61:54–64.

[37] FitzGerald M, Crowley T, Greenhouse P, Probert C, Horner P. Teaching sexual history taking to medical students and examining it: experience in one medical school and a national survey. Med Educ. 2003;37(2):94–8. 12558878 10.1046/j.1365-2923.2003.01411.x

[38] Dietrich E, Le Corre Y, Dupin N, Dréno B, Cartier I, Granry JC, et al. Benefits of simulation using standardized patients for training dermatology residents in breaking bad news. Ann Dermatol Venereol. 2021;148(3):156–60. 33487487 10.1016/j.annder.2020.11.003

[39] Bagacean C, Cousin I, Ubertini AH, et al. Simulated patient and role play methodologies for communication skills and empathy training of undergraduate medical students. BMC Med Educ 2020;20:491. 10.1186/s12909-020-02401-0. 10.1186/s12909-020-02401-0 PMC7716460 33276777

[40] Romme S, Bosveld MH, Van Bokhoven MA, De Nooijer J, Van den Besselaar H, Van Dongen JJJ. Patient involvement in interprofessional education: A qualitative study yielding recommendations on incorporating the patient's perspective. Health Expect. 2020;23(4):943-957. 10.1111/hex.13073. Epub 2020 Jun 4. PMID: 32496648; PMCID: PMC7495081. 10.1111/hex.13073 PMC7495081 32496648

[41] Sullivan C, Doyle AJ, O'Toole M, Mulhall C, McNaughton N, Eppich W. 'How can we help the students learn?' A grounded theory study of simulated participants as educators. Med Teach. 2023;45(9):1047–53. 10.1080/0142159X.2023.2171857. Epub 2023 Feb 1. PMID: 36726233. 10.1080/0142159X.2023.2171857 36726233

[42] Pritchard SA, Blackstock FC, Keating JL, Nestel D. The pillars of well-constructed simulated patient programs: A qualitative study with experienced educators. Med Teach. 2017;39(11):1159–67. 10.1080/0142159X.2017.1369015. Epub 2017 Aug 28. PMID: 28845722. 10.1080/0142159X.2017.1369015 28845722

[43] Uppor W, Klunklin A, Viseskul N, Skulphan S. Effects of experiential learning simulation-based learning program on clinical judgment among obstetric nursing students. Clin Simul Nurs. 2024;92:101553.

[44] Mattout SK, Shah B, Khan MM, Mitwally N, Aseri ZA, Yousef EM. Realistic simulation case scenario as a vertical integration teaching tool for medical students: a mixed methods study. JTUMED [Internet]. 2023;18(6):1536–44. 10.1016/j.jtumed.2023.08.001. 10.1016/j.jtumed.2023.08.001 PMC10494172 37701845

[45] Lovink AG, Groenier M, Niet A, van der, Miedema H, Rethans JJ. The contribution of simulated patients to meaningful student learning. Perspect Med Educ. 2021;10(6):343–50. 10.1007/s40037-021-00684-7 PMC8633349 34637120

[46] Smith CM, Gliva-McConvey G, Chapin A. Training SPs for authentic role portrayal. In: Comprehensive healthcare simulation: Implementing best practices in standardized patient methodology. Cham: Springer International Publishing; 2020. p. 73–104.

[47] Clarke V, Braun V. Thematic analysis. J Posit Psychol. 2017;12(3):297–8.

[48] Lenhart C, Bouwma–Gearhart J. Engaging students around the complex socioscientific issue of sustainability: affordances and tensions of faculty working across disciplines to develop transdisciplinary curricula. CBE- Life Sciences Education. 2022. 10.1187/cbe.21-03-0075. 10.1187/cbe.21-03-0075 PMC9508926 35580003

[49] Albert JS, Younas A, Sana S. Nursing students’ ethical dilemmas regarding patient care: an integrative review. Nurse Educ Today. 2020;88:104389. 32193068 10.1016/j.nedt.2020.104389

[50] Yee M, Nyunt M, Thidar A, Khine M, Ong C, Seong O. Challenges and opportunities of using role-players in medical education: medical educator’s perspective. Med Res Arch. 2024;12(8) https://www.esmed.org/MRA/mra/article/view/5582.

[51] Nestel D, Tabak D, Tierney T, et al. Key challenges in simulated patient programs: An international comparative case study. BMC Med Educ. 2011;11:69. 10.1186/1472-6920-11-69. 10.1186/1472-6920-11-69 PMC3189900 21943295

[52] Aiello MT, Struijk JS, Szauter K, Nicholas CF. (2020). Professional Development of the SP Educator. In: Gliva-McConvey G, Nicholas CF, Clark L. (eds). Comprehensive Healthcare Simulation: Implementing Best Practices in Standardized Patient Methodology. Comprehensive Healthcare Simulation. Springer, Cham. 10.1007/978-3-030-43826-5_11.

