# Supplementary material for: Validating simulated patient programmes in Obstetrics and Gynaecology education: a mixed-method study on training effectiveness and stakeholder perceptions in the GCC
Source: BMC Med Educ. 2025 Oct 17;25:1439. doi: 10.1186/s12909-025-07912-2 (PMC12532415; doi:10.1186/s12909-025-07912-2)
Supplement: Supplementary file 1 — Supplementary Material 1. [file 12909_2025_7912_MOESM1_ESM.pdf]

Simulated Patient – Project (Data Collection Sheet)

Form 1

Scenario 1: Early pregnancy bleeding

Research ID of the student: **Date:** **Gender: Male / Female**

OSCE CHECKLIST & MARKING SHEET

**Focused history taking station**	**Missed (0)**	**Poor (1)**	**Fair (2)**	**Satisfactory (3)**	**Outstanding (4)**
**Present illness**<br>• color of the bleeding, amount, frequency<br>• Is it associated with pain?<br>• Is there history of passing tissue, fever, bad vaginal odor?<br>• Is there history of passing vesicles, excessive nausea and vomiting?<br>• Is there history of post coital bleeding?<br>• Any h/o fainting?					
**Current obstetric history**<br>• Last menstrual period (LMP)<br>• Expected date of delivery (EDD)<br>• Blood tests, Ultrasonogram<br>• Weight gain, other problems during pregnancy (gestational diabetes mellitus, blood pressure, fever / Infection)					
**Past obstetric history**<br>• Gravidity, parity, miscarriages, duration of pregnancy outcome, complications					

Simulated Patient – Project (Data Collection Sheet)

**Gynecology history**<br>• Menstrual history (H/O first menarche regularity, duration, amount, associated with pain)<br>• H/O vaginal discharge, contraception					
**Past medical & surgical history**:<br>• H/O hypertension, renal diseases, autoimmune disease, diabetes mellitus, bronchial asthma, any other diseases<br>• H/O previous surgery					
**Family history & social history**<br>• H/O hypertension, diabetes mellitus, or any other disease in the family<br>• H/O smoking / alcohol					
**Professionalism & communication**<br>• Greets the patient and introduces self<br>• Allows the patient to complete his/her opening statement (story) without interruption<br>• Listens attentively to the patient<br>• Elicits patient's concerns and beliefs<br>• Responds explicitly to patient's queries<br>• Summarizes and checks patient's understanding					
**Total marks**					

Signature of the faculty
