# Supplementary material for: Validating simulated patient programmes in Obstetrics and Gynaecology education: a mixed-method study on training effectiveness and stakeholder perceptions in the GCC
Source: BMC Med Educ. 2025 Oct 17;25:1439. doi: 10.1186/s12909-025-07912-2 (PMC12532415; doi:10.1186/s12909-025-07912-2)
Supplement: Supplementary file 2 — Supplementary Material 2. [file 12909_2025_7912_MOESM2_ESM.pdf]

Simulated Patient – Project (Data Collection Sheet)

Form 2

Scenario 2: Pre-eclampsia

Research ID of the student: **Date:** **Gender: Male / Female**

OSCE CHECKLIST & MARKING SHEET

**Focused history taking station**	**Missed (0)**	**Poor (1)**	**Fair (2)**	**Satisfactory (3)**	**Outstanding (4)**
**Chief complaints (**onset, duration, intensity)<br>• Headache<br>• Blurred vision/flushing lights<br>• Irritability<br>• H/O high blood pressure<br>• H/O proteinuria<br>• H/O edema					
**Associated symptoms** & **Complications** (onset, duration, intensity)<br>• Right upper quadrant pain<br>• Nausea, vomiting<br>• Fetal movement<br>• Per vaginal bleeding<br>• Breathing difficulties<br>• Convulsion/eclampsia fits					
**Obstetric history (current)**<br>• Last menstrual period (LMP)<br>• Expected date of delivery (EDD)<br>• Blood tests, ultrasonogram, weight gain<br>• Other problems during pregnancy (gestational diabetes mellitus, vaginal discharge, fever / infection)					

**Past obstetric history**<br>• Gravidity, parity, miscarriages, duration of pregnancy outcome, complications					
**Past medical & surgical history**:<br>• H/O Hypertension, renal diseases, autoimmune disease, diabetes mellitus, bronchial asthma, any other diseases<br>• H/O Previous surgery					
**Menstrual history, family history & social history**<br>• Menstrual history (H/O first menarche regularity, duration, amount, associated with pain)<br>• H/O vaginal discharge, contraception<br>• H/O hypertension, diabetes mellitus, or any other disease in the family<br>• H/O smoking / alcohol					
**Professionalism & communication**<br>• Greets the patient and introduces self<br>• Allows the patient to complete his/her opening statement (story) without interruption<br>• Listens attentively to the patient<br>• Elicits patient's concerns and beliefs<br>• Responds explicitly to patient's queries<br>• Summarizes and checks patient's understanding					
**Total marks**					

Signature of the faculty
