# Supplementary material for: Validating simulated patient programmes in Obstetrics and Gynaecology education: a mixed-method study on training effectiveness and stakeholder perceptions in the GCC
Source: BMC Med Educ. 2025 Oct 17;25:1439. doi: 10.1186/s12909-025-07912-2 (PMC12532415; doi:10.1186/s12909-025-07912-2)
Supplement: Supplementary file 3 — Supplementary Material 3. [file 12909_2025_7912_MOESM3_ESM.pdf]

<u>Form 5</u>

**ID of the SP:**	**ID of the student:**	**Gender of the student: Male / Female**	**Name of the scenario:**

Evaluation of SP performance / SP program by students (Kindly rate as per the given scale)

**Parameter**<br>During the encounter I felt that the SP:	**Strongly Disagree (1)**	**Disagree (2)**	**Neutral (3)**	**Agree (4)**	**Strongly agree (5)**
Has the ability to fit in to the role					
Has ability to communicate well with me					
Played his / her role in a professional manner					
Gave me sufficient time to talk (e.g., didn't interrupt).					
Listened to me carefully					
Gave me undivided attention					
Answered all my questions with relevant answers					
Clarified information whenever I asked for					
Maintained a respectful tone (e.g., did not belittle me; did not talk down to me).					
Made me feel comfortable during the course of the encounter					
Asked me leading questions and guided me through the process					
I would like to be trained with SPs for other scenarios as well					
I would recommend SPs for regular training in all clinical departments					

What one thing you **liked the most** about the SP interaction?
What one thing you **did not like** about the SP interaction?
What could have been done **better** in the SP interaction?
What are your **suggestions** for the improvement of the SP program?
