# Supplementary material for: Validating simulated patient programmes in Obstetrics and Gynaecology education: a mixed-method study on training effectiveness and stakeholder perceptions in the GCC
Source: BMC Med Educ. 2025 Oct 17;25:1439. doi: 10.1186/s12909-025-07912-2 (PMC12532415; doi:10.1186/s12909-025-07912-2)
Supplement: Supplementary file 4 — Supplementary Material 4. [file 12909_2025_7912_MOESM4_ESM.pdf]

Form 6

Evaluation of SPs' performance by Raters

Kindly rate as per the given scale.

Needs improvement (1), Average (2), Good (3)

**During the student – SP encounter I observed that the SP has:**	**Rating**
Ability to fit in the role	
Ability to communicate with the students	
Ability to complete the History	
Ability to observe the scenario and reflect	
Ability to show emotions needed for the scenario	
Ability to ask the given questions in the sequence	
Ability to maintain standardization	

Other Comments: --------

Signature of the rater: -------
