# Supplementary material for: Validating simulated patient programmes in Obstetrics and Gynaecology education: a mixed-method study on training effectiveness and stakeholder perceptions in the GCC
Source: BMC Med Educ. 2025 Oct 17;25:1439. doi: 10.1186/s12909-025-07912-2 (PMC12532415; doi:10.1186/s12909-025-07912-2)
Supplement: Supplementary file 5 — Supplementary Material 5. [file 12909_2025_7912_MOESM5_ESM.pdf]

Form 3

Evaluation of students' performance by SPs

Kindly rate as per the given scale:

Strongly Disagree (1), Disagree (2), Neutral (3), Agree (4)   Strongly agree (5)

**During the encounter I felt that the student:**	**Rating**
Made a personal connection with me (e.g., wished me, smiled at me, went beyond medical issues at hand, conversed about personal background, interests, job, etc.)	
Gave me an opportunity/time to talk (e.g., didn't interrupt)	
Listened to me, gave me undivided attention (e.g., eye contact, verbal acknowledgment, non-verbal feedback).	
Checked/clarified information (e.g., recapped, paraphrased, echoed, summarized, asked for clarification when not certain about what I described)	
Encouraged me to ask questions/checked my understanding of information provided.	
Adapted to my level of understanding, using appropriate language (e.g., avoided or explained jargon: avoided child-like slang).	
Expressed empathy (e.g. demonstrated care and concern for me, acknowledged my feelings, expressed understanding of my feelings/ respect for my situation/ willingness to support me).	
Maintained a respectful tone (e.g, did not belittle me; did not use humour inappropriately, did not talk down to me).	
Involved me in deciding upon a plan (e.g., presented me with options (if any), provided rationale for their assessment)	
Elicited and addressed any concerns I have about the plan.	
Did you ever feel uncomfortable during the course of the encounter?	**Yes / No**
If yes, please comment. Please describe when this occurred, what specific behaviour led to this feeling, and how you felt	
Did the student ever appear uncomfortable during the course of the encounter?	**Yes / No**
If yes, please comment. Please describe when this occurred, what specific behaviour led to this feeling, and how you felt	

Other Comments: --------

Signature of the SP: -------
