# Supplementary material for: Validating simulated patient programmes in Obstetrics and Gynaecology education: a mixed-method study on training effectiveness and stakeholder perceptions in the GCC
Source: BMC Med Educ. 2025 Oct 17;25:1439. doi: 10.1186/s12909-025-07912-2 (PMC12532415; doi:10.1186/s12909-025-07912-2)
Supplement: Supplementary file 6 — Supplementary Material 6. [file 12909_2025_7912_MOESM6_ESM.pdf]

Form 7

Evaluation of SP program by SPs

Kindly rate as per the given scale.

Strongly Disagree (1), Disagree (2), Neutral (3), Agree (4)   Strongly agree (5)

**As SP, I feel that**	**Rating**
The SP training is structured / organized properly	
I am happy about the way the program is going on	
I do not have difficulty in understanding the scenarios	
I receive adequate information and training beforehand	
The students are cooperative and respectful	
It is convenient for me to work as SP	

Other Comments: --------

Signature of the SP: -------
