# Supplementary material for: Validating simulated patient programmes in Obstetrics and Gynaecology education: a mixed-method study on training effectiveness and stakeholder perceptions in the GCC
Source: BMC Med Educ. 2025 Oct 17;25:1439. doi: 10.1186/s12909-025-07912-2 (PMC12532415; doi:10.1186/s12909-025-07912-2)
Supplement: Supplementary file 7 — Supplementary Material 7. [file 12909_2025_7912_MOESM7_ESM.pdf]

MEDICAL SKILLS AND SIMULATION CENTER (MSSC)

ARABIAN GULF UNIVERSITY

REGISTRATION FORM FOR SIMULATED PATIENT (SP)

Name: ______________________________

Date of Birth: ______________________________

Nationality: ______________________________

Contact: Mobile 1. ______________ Mobile 2. ______________

E-mail: ______________________________

Address (Home): ______________________________

Postal Address: ______________________________

Occupation: ______________________________

Previous Experience: ______________________________

Time of Availability to work in MSSC:

____

Please tick (√)

- ☐ Can come once daily
- ☐ Can come once weekly
- ☐ Can come on monthly basis once or twice

Type of Work:

I can have the work as

a. Talking to Students or Doctors only.
b. Talking and allow students to examine my lung, heart & check BP or abdomen.

Payment:

I accept working on temporary basis & get my salary on hourly or session basis every month, according to the total number of hours I worked in AGU.

Signature of SP

____

Date:

Signature of SP Coordinator

____

Date:

Signature of MSSC Program Director

____

Date:
