# Supplementary material for: Validating simulated patient programmes in Obstetrics and Gynaecology education: a mixed-method study on training effectiveness and stakeholder perceptions in the GCC
Source: BMC Med Educ. 2025 Oct 17;25:1439. doi: 10.1186/s12909-025-07912-2 (PMC12532415; doi:10.1186/s12909-025-07912-2)
Supplement: Supplementary file 9 — Supplementary Material 9. [file 12909_2025_7912_MOESM9_ESM.pdf]

Validating Simulated Patient Programs in OB-GYN Education: A Mixed Methods Study on Training Effectiveness and Stakeholder Perceptions

Results

Socio-demographic data of Participants

Findings from thematic analysis

Theme 1: Standardised patient program as a comparable educational strategy for clinical competencies development

1.1 Address ethical and social challenges associated with practicing on real patients

Participants reported that direct exposure to real patients during clinical postings comes with various challenges. The shorter hospital stays and improvement in healthcare has reduced the patient availability, and reduced opportunities for medical students to interact with hospitalized patients. Additionally, increasing awareness of patients' rights, confidentiality and medical ethics further decline the clinical exposure of students. Furthermore, patients' hesitation and refusal to cooperate, particularly while experiencing pain or anguish or discussing sensitive themes, appeared as a major barrier to communication. Variability in patient responses or sharing of irrelevant information by the patients due to recall issues, subjective nature of pain or symptoms or misunderstanding of questions results in confusion and additional cognitive load for students. This makes it difficult for students to focus on key details required for making accurate diagnosis. Given these challenges, the participants highlighted the need for structured training through simulated patients to ensure that student receive consistent case presentation allowing them to practice in structured manner while mitigating the issues associated with real patients.

1.2 Provide safe and structured space for learning

All participants, including trainers, SPs, leaders, and students, agreed that training with SPs provides a realistic but risk-free environment in which students may repeatedly practice history taking, physical examination, and patient communication in a systematic manner. This experience allows them to make mistakes, gain feedback from the trainers, and fine-tune their skills before working with actual patients. They felt that this experience minimizes the likelihood of medical errors while also ensuring the safety of real patients through improved hands-on experience and confidence. Furthermore, participants said that training with SPs

allowed students to practice rapport building, professional behavior, and empathy. It also teaches them how to conduct sensitive medical discussions with patients when they begin working in clinical settings.

1.3 Opportunity for Standardised and equal exposure to multiple health conditions

All participants agreed that, unlike real patients whose availability and medical conditions are unpredictable, the SP program exposes students to a wide range of health issues that are difficult to encounter in real clinical settings. The SPs provided scripts that simulate a range of medical problems from several fields, allowing students to gain experience focusing on varied scenarios.

Moreover, working with SPs helps to resolve inequities in clinical exposures, ensuring that all students have an equal opportunity to practice and improve their abilities. In real life, exposure to such crucial learning situations is frequently limited by patient availability and students' access to various departments, particularly the psychiatric, gynecological, obstetric, and oncology departments, where dealing with such patients could be challenging.

Theme 2: Challenge associated with SP Program

2.1 Lack of realism and authenticity

Participants believed that familiarity with the SP influenced the authenticity of practicing clinical skills. The SP asserted that students fail to take them seriously since they are performers rather than actual patients. This results in a lack of engagement with the case. In addition, students also noted that encounters with SP frequently lack the patients' actual unpredictability and emotions due to weak acting abilities. As a result, they have difficulty immersing themselves in the circumstances. Another difficulty affecting authenticity was that while SPs with medical or nursing backgrounds make it easy to understand the diagnosis, they struggle to adequately mimic the behavioral diversity of genuine cases, limiting their real-world usefulness. As a result, almost all participants believed that SP could not totally replace real-life patient interactions since some medical conditions, emotional responses, and physiological changes can only be experienced by real patients.

2.2 Challenge in recruitment and retention of SPs

In terms of recruitment, the program leaders reported difficulty in recruiting SPs due to various reasons. One major challenge is gender barrier. Particularly, women don't appear for SPs interview as they do not want to get examined by male students. Language barrier further complicates the recruitment procedure as there is diversity in slangs or dialects across regions, which make it difficult for trainers to teach SPs use appropriate medical terminology or phrasing during the sessions. Besides, a general lack of awareness in the community among SP program and its benefit for healthcare, thus many people feel hesitant to apply for SP positions.

In terms of retentions, conflicting schedule and absenteeism during the training program was noted as key challenge in long term retention of SPs. Since SPs have other jobs or commitments, they find it difficult to accommodate training and exam schedules, especially when sessions are scheduled on short notice. On the other hand, some of the SPs acknowledges that they often discontinue the program due to less payment they receive for the work or due to feeling of burnout and fatigue after appearing in multiple sessions.

2.3 Challenges associated with program designing, standardization and implementation

The problem of SP recruitment and retention was noted to have an impact on program design and delivery. Trainers and leaders are frequently required to change the training program based on the availability of the SPs. Likewise, absenteeism among SPs was reported to cause last-minute confusion and substitutions, affecting the continuity or consistency of the training programs for students. Furthermore, time limits, role complexity, difficulties in emotional expression, and confidentiality issues were cited as barriers to designing, standardizing, and implementing the SP program. Both SPs and trainers agree that the limited amount of time given for simulation preparation is insufficient to effectively prepare for the role. Furthermore, many SPs feel pressured to memorize long scripts in such a short period of time, jeopardizing their ability to maintain consistency during role play. This effects their emotional portrayal of the scripts. Some trainers claimed that SPs struggle to relate to complex topics and hence cannot represent emotions such as pain or despair, making them unrealistic for students.

Maintaining confidentiality of role was noted as another challenge. Trainers and leaders stated that they are not allowed to discuss scripts with SPs ahead of time in order to ensure fair assessment of students during clinical practice or exams. However, the SP disclosed that despite being aware of the value of confidentiality, they've experienced an identity conflict between their obligations as an SP and as parents. As a result, some of them rarely violate exam confidentiality by disclosing the diagnosis/provide additional information to students.

Theme 3: Suggestion for Improving SP program

3.1 Improving SP working conditions

Better payment was identified as a crucial motivator, with participants indicating that an increase in income would increase their motivation and commitment to continue working at the institution. Expanding the pool of SPs and offering opportunities to participate in varied roles through cross-collaboration and exchanging SPs across institutions was also proposed as a way to prevent SP burnout and financial strain on institutions. Furthermore, breaks between long sessions were recommended to preserve focus and persistence. Clear and structured scenario guidelines, as well as faculty engagement in training, were suggested to improve SPs' case presentation.

3.2 Enhancing program accessibility and flexibility

Participants recognized that in order to improve the accessibility and flexibility of the SP program, the schedule of trainings should be stated in advance using accessible media/forums such as a WhatsApp group to ensure attendance at the sessions. Furthermore, hybrid training sessions should be held at regular intervals to ensure participation. Furthermore, participants indicated that in order to reduce absenteeism during the sessions, backup SPs should be kept to ensure session consistency.

3.3. Expanding and strengthening training

All participants agreed on the importance of having planned and regular training sessions with SPs at regular intervals, as well as mock training sessions for students throughout the year, to reinforce the training program. They envisioned that it would assist the SPs polish their acting

skills while also improving the students' professional skills. Furthermore, participants stated that clinical faculty members should be invited to assist with understanding complex concepts in the scripts, as well as during SP training sessions to provide feedback on the scripts and case presentation.

Participants also noted that adopting more stringent selection criteria for SP, such as individuals with an acting experience, some previous knowledge of medical fields, having an existing illness or being bilingual, agree to strictly adhere to maintaining confidentiality may contribute to improving the authenticity and engagement of learners in SP training programs. They also underline the need to train clinical faculty in script writing for clinical scenarios.

Theme	*Sub-themes*	*Codes*	*Meaning quotes*
**Simulated patient program as a comparable educational strategy for clinical competencies development**	**Address ethical and social challenges associated with practicing on real patients**	• Shorter hospital stays<br>• Privacy and ethical concerns<br>• Gender barrier<br>• Irrelevant information sharing	*"Many students want to take history but they don't have access to many patients as in the modern medical care, we don't admit patients for a long time. Many patients don't want to be disturbed and maintain their confidentiality. With growing awareness of Patient rights, Ethics, it is becoming difficult for our students to practice any clinical skill on real patients." [Leadership 3]*<br><br>*"Some patients may not talk to the opposite gender freely and especially in history related to Mental health, Sexual health or gender related concerns. [Leadership* 2]<br><br>*when we usually go to take history from real patients, they sometimes respond something which is irrelevant to their case, that affect our history taking and the provisional diagnosis."[Student 3]*
	**Provide safe and structured space for learning**	• Realistic yet risk-free environment<br>• Provide room for mistakes<br>• Minimizes the likelihood of medical errors<br>• Learn skills of sensitive medical discussions	*"I think having simulated patients for training is a very good initiative. You know, they let us practice things like clinical skills and having sensitive or tough conversations without actually …worrying about actually hurting anyone or fear of getting judged by others. It's kinda like building confidence in a safe space"* [QA_Student 6]<br><br>*"SPs can be used for very difficult conversations, such as breaking up bad news, sexual history, menstrual history, cancer history, and HIV history, which most students might find difficult to get a chance to discuss in the real world."* [QA_SP_4]
	**Opportunity for Standardised and equal exposure to multiple health conditions**	• Exposure to wide range of health issues.<br>• resolve inequities in clinical exposures.	*"They get exposed to different types of cases and personalities. Do you think you will get So many cases of Type 2 diabetes with Infected wound on the same day for all the students. No, it's not possible. similarly, the case of Mania or a case of depression? So, it's really important the college takes more steps" [QA_SP 2]*<br><br>*"All students get the same opportunity. And I think it makes their exam more Standardized. It also help us to create rare cases, Difficult scenarios, which the student may see in the future, cases that are not available in this country, but they may be seen in other countries" [QA_SP_3]*

**Challenge associated with SP Program**	**Lack of realism and authenticity**	• Identify as performer rather than patient<br>• SPs lack the patients' actual unpredictability and emotions<br>• SPs lack behavioural diversity of genuine cases	*"Because we they would have seen us here many a times and they know that we are actors and we are not real patients. So some of them don't take us seriously" [QA_SP_ 1]*<br><br>*"Like, first, sometimes it doesn't feel totally real, you know? No matter how well they act, there's still that thought in the back of your mind that this isn't a real patient, so the experience isn't always 100% realistic." [*QA_Student 6]<br><br>*OK, we had few nurses who were acting as SPs. That nurses already have medical background. At the same time, so it might be, not be exactly the same level as you see in real patients in a hospital or a Health Center." [QA_Trainer 6]*
	**Challenge in recruitment and retention of SPs**	• Gender barrier<br>• Language barrier<br>• Lack of community awareness about SP program<br>• conflicting schedule and absenteeism<br>• Low Pay<br>• Fatigue and burnout	*I think in our culture, especially in GCC like Bahrain, people cannot understand the concept of SP. They are afraid or reluctant to act especially with male students. Many people, they don't understand why it is relevant and beneficial to healthcare." [QA_Trainer 1]*<br><br>*"We are expected to stay here for six hours or so during exams. All of us work elsewhere as part timers. So, we have to plan our schedules accordingly."[QA_SP_ 1]*<br><br>*"I think money also plays a part. Many SP's feel the fees what we are getting is not sufficient so they don't really want to get involved in the program. Also, It is tiring for SPs to repeat the same scenario multiple times. " [QA_SP_3]*
	**Challenges associated with program designing, standardization**	• Difficulty in aligning schedule<br>• Absenteeism disrupts program consistency	*Also, our schedule has to align with their schedule. They usually prefer to come in the afternoon or towards the end of the day. But our clinical training starts in the morning. So, we have to adjust that schedule. [QA_Leadership 4]*<br><br>*Absenteeism is one problem. for example, we arranged exam for today. Some SP's all of a sudden in the morning will tell like, 'today I can't come'. The trainers get*

	**and implementation**	• Restricted time for role practice<br>• Role complexity<br>• Difficulties in emotional expression,<br>• Confidentiality issues	*tense. They run here and there; the entire session gets disrupted, even delaying some of the exams by 15 minutes. Half an hour." [QA_Trainer 10]*<br>*"The time given for training is very short- only a few minutes, so it really puts lots of pressure on us to fully prepare for certain roles and affect the quality of the training we provide." [QA_SP 2]*<br>*I think one difficulty that I experienced with SP training is that their inability to relate to the situation. They don't have experience to act based on the script that was provided to them., so when they are supposed to act, as they have real pain. But they act very normal for a painful script also."[QA_Trainer 9]*<br>*"Even though, we should not discuss these things with students. Some of my colleagues feel that we have to help them. Our children also going to college. We know how they struggle. They try to help them by revealing the diagnosis or giving more hints during the exam [QA_SP 2]*
**Suggestion for Improving SP program**	**Improving SP working conditions**	• Better Pay<br>• SP exchange across institutions<br>• Break between schedules<br>• Simple and clear role play guidelines	*If we increase the amount…I think we can get more commitment and motivation from SPs." [QA_Trainer 8]*<br>*"There are two, three colleges, it will be better if they can share SPs so that you know the money involved in it gets shared between the institutions. Thank you."[QA_SP_3]*<br>*"Scheduling breaks between the session is very important. Unfortunately, during exams we sit for a long time and it leads to burnout. It will be nice if they can give us a break after every six student or seven students. You know, I know it's difficult, but it's. How we feel also,"[QA_SP_3]*<br>*"All trainers should be given clear guide that that information should be conveyed to the SP. that those are the red flags that we should convey to that SP, and those are the crucial points that he should not say or she should not say."[QA_Trainer 3]*
	**Enhancing program accessibility and flexibility**	• Back – ups<br>• Forum for SP coordination<br>• Hybrid training sessions	*So we should have somebody back up. Also, I think we should have common whatsapp group and you know contact them. Because personal messaging is difficult. We need to have separate forum, for SP coordination, and to disseminate some common information."[QA_Trainer 10]*<br><br>*"Some sessions can be online …some face to face. We should prepare our SPs for those scenarios…for two days at least." [QA_Trainer 8]*

	**Expanding and strengthening training**	• planned and regular training sessions with SPs<br><br>• Regular Mock training sessions for students<br><br>• Inclusion of Clinical faculties in training sessions<br><br>• Strict SP recruitment criteria	*I think regular, spaced training sessions would help a lot. Instead of just cramming for exams or focusing only on simulation centers, we could have ongoing sessions throughout the year to keep practicing, not just professional skills but communication, and empathy, also. It'd feel more natural and like real patient interactions." [QA_Student 6]*<br><br>*"We strongly believe having a clinical faculty during the training and validation of the SP adds more value to the team as well, because even though our trainers are good they are not clinicians. They do not know the nuances while training our SPs" [QA_Leadership 3]*<br><br>*"I recommend that some clinicians or faculty who write the script, should help us in the training, especially if it contains difficult terms."*[QA_Trainer 7]<br><br>*" We must training our clinical faculty on how to simplify the scenario. How to know what they expect? What emotion they have to display and what should be SP's body language etc. should be clearly written in the script. "[QA_Leadership 3]*<br><br>"*So, we will try to get more educated, Bilingual candidates who are motivated and can act and if they have some disease entity that can be used, such as hypertension or diabetes" [QA_Leadership 1]*<br><br>*"Colleges can recruit SP from Nurses or supporting staff, who are close to healthcare, or It will be better if we get an SP with an acting background like me. "[QA_SP_4]*

Figure 1: Word Cloud of Codes

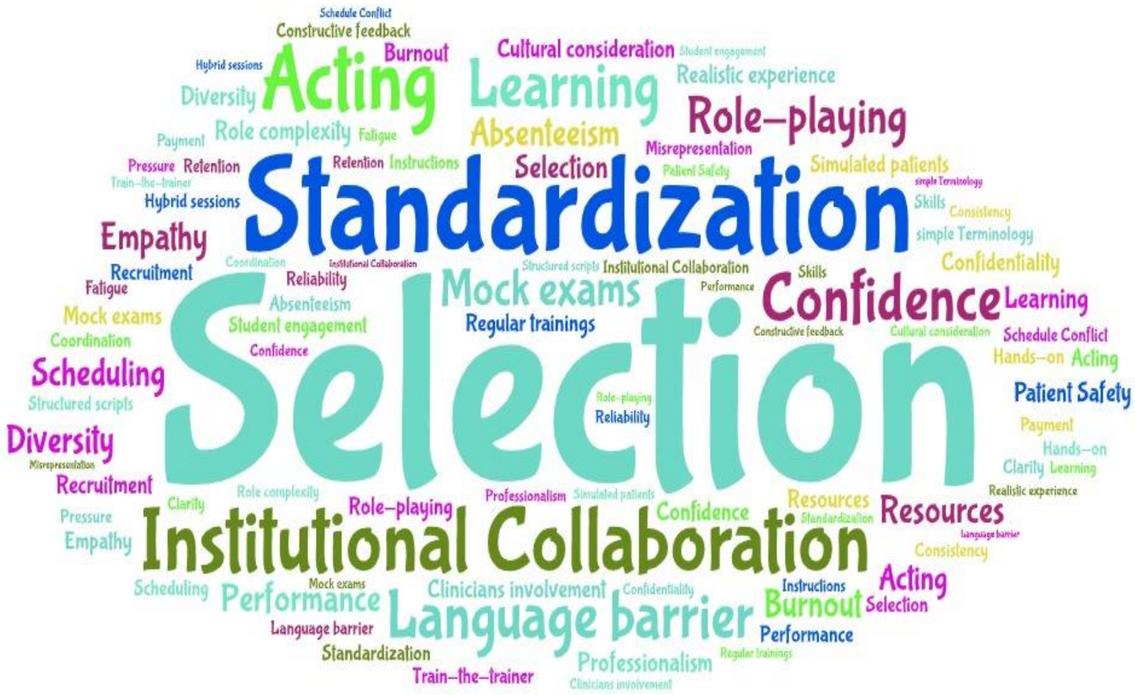

Methodology

All audio recordings of interviews were thoroughly transcribed and translated into English by the two authors who originally conducted the interviews. Another expert assessed the transcripts to confirm that the translation process was accurate. The participants' identifying information was omitted and the conversations were assigned based on their designation/role, such as student, leader, trainer, SP (simulated patient). All of the transcripts were subsequently imported into the MAXQDA software (24 Analytic Pro trial version) for data sorting. A thematic analysis with an inductive method was used to generate codes and themes. We used the Braune and Clarke approach for thematic analysis. The first step was to thoroughly read the transcripts multiple times in order to become immersed in the data. The meaning units of data were then extracted line by line. Each meaning unit was simplified into codes, which captured the individuals' unique experiences. Subsequently, all codes were rigorously organized and merged into potential sub-themes and overarching themes. Two authors refined the sub-themes and themes several times to ensure that they appropriately expressed the phenomenon of interest. Any disagreements between the authors were handled by mutual conversation.
