# Supplementary material for: Validating simulated patient programmes in Obstetrics and Gynaecology education: a mixed-method study on training effectiveness and stakeholder perceptions in the GCC
Source: BMC Med Educ. 2025 Oct 17;25:1439. doi: 10.1186/s12909-025-07912-2 (PMC12532415; doi:10.1186/s12909-025-07912-2)
Supplement: Supplementary file 10 — Supplementary Material 10. [file 12909_2025_7912_MOESM10_ESM.pdf]

Interview guide – Leadership

Introduction
Greetings!. I am Dr Archana Prabu Kumar, Assistant Professor, Medical Education Department, CMMS, AGU.

Title: Training and Standardization of Simulated Patients for Teaching History Taking Skills to Medical Students. I am the principal investigator of this study, which is approved by REC, CMMS, AGU (E013-PI-11/19).
As part of this study, we invite you to participate in this discussion and share your views. This discussion is audio recorded for the purpose of data analysis. We assure you that all the data (including your identity) will be kept confidential. You have the right to refuse and your participation in this study is entirely voluntary, and your consent is required before you can participate in this study.

Leadership:
Can you ***describe*** as a Dean / Director / Chairperson, regarding the experience faced by the students when they try to take history from 'real' patients in hospitals / community health centers (with special focus on challenges)

In your opinion, do you think that there is a ***need to introduce*** 'simulated patients' as part of regular clinical training? If yes, give reasons for your answer.

In your opinion, ***what aspects*** (history taking / clinical examination / counselling etc) of medical training, should involve 'simulated patients'?

In your opinion, what are the disciplines (internal medicine, surgery, OG etc) of medical training, should involve 'simulated patients'?

In your opinion, what are the ***advantages*** of introducing 'simulated patients' in medical training,

In your opinion, what are the ***potential challenges*** in introducing 'simulated patients' in medical training?

Can you share few ***suggestions***, to overcome the challenges in introducing 'simulated patients' in medical training?

Thank you for participating in this research study

Interview guide – Students

Introduction
Greetings!. I am Dr Archana Prabu Kumar, Assistant Professor, Medical Education Department, CMMS, AGU.

Title: Training and Standardization of Simulated Patients for Teaching History Taking Skills to Medical Students. I am the principal investigator of this study, which is approved by REC, CMMS, AGU (E013-PI-11/19).
As part of this study, we invite you to participate in this discussion and share your views. This discussion is audio recorded for the purpose of data analysis. We assure you that all the data (including your identity) will be kept confidential. You have the right to refuse and your participation in this study is entirely voluntary, and your consent is required before you can participate in this study.

Students:

Can you ***describe your experience*** while taking history from ‘real’ patients in hospitals / community health centers (with special focus on challenges)

In your opinion, do you think that there is a ***need to introduce*** ‘simulated patients’ as part of your regular clinical training? If yes, give reasons for your answer.

In your opinion, ***what aspects*** (history taking / clinical examination / counselling etc) of medical training, should involve ‘simulated patients’?

In your opinion, what are the disciplines (internal medicine, surgery, OG etc) of medical training, should involve ‘simulated patients’?

In your opinion, what are the ***advantages*** of introducing ‘simulated patients’ in medical training,

In your opinion, what are the ***potential challenges*** in introducing ‘simulated patients’ in medical training?

Can you share few ***suggestions***, to overcome the challenges in introducing ‘simulated patients’ in medical training?

Thank you for participating in this research study

Interview guide – Simulated Patients

Introduction
Greetings!. I am Dr Archana Prabu Kumar, Assistant Professor, Medical Education Department, CMMS, AGU.

Title: Training and Standardization of Simulated Patients for Teaching History Taking Skills to Medical Students. I am the principal investigator of this study, which is approved by REC, CMMS, AGU (E013-PI-11/19).
As part of this study, we invite you to participate in this discussion and share your views. This discussion is audio recorded for the purpose of data analysis. We assure you that all the data (including your identity) will be kept confidential. You have the right to refuse and your participation in this study is entirely voluntary, and your consent is required before you can participate in this study.

Simulated Patients:

Can you ***describe your experience*** while students take history from 'you or when they examine you?

In your opinion, do you think that there is a ***need to introduce*** 'simulated patients' as part of student medical training? If yes, give reasons for your answer.

You would have played several roles as SP, In your opinion, ***which skills / tasks*** (history taking / clinical examination / counselling etc), are more easier for you to get trained as 'SPs'. Give reasons for your answer.

You would have participated in several exams representing many departments / disciplines In your opinion, ***what are the disciplines*** (internal medicine, surgery, OG etc) that are more easier for you to get trained as 'SPs'. Give reasons for your answer.

In your opinion, what are the ***advantages*** of introducing 'simulated patients' in medical training,

In your opinion, what are the ***potential challenges*** in introducing 'simulated patients' in medical training? You can describe from your own experience.

Can you share few ***suggestions***, to overcome the challenges in introducing 'simulated patients' in medical training?

Thank you for participating in this research study

Interview guide – Faculty Trainers

Introduction
Greetings!. I am Dr Archana Prabu Kumar, Assistant Professor, Medical Education Department, CMMS, AGU.

Title: Training and Standardization of Simulated Patients for Teaching History Taking Skills to Medical Students. I am the principal investigator of this study, which is approved by REC, CMMS, AGU (E013-PI-11/19).
As part of this study, we invite you to participate in this discussion and share your views. This discussion is audio recorded for the purpose of data analysis. We assure you that all the data (including your identity) will be kept confidential. You have the right to refuse and your participation in this study is entirely voluntary, and your consent is required before you can participate in this study.

Trainer / faculty:

- Can you ***describe your experience*** while training / recruiting simulated patients (SPs). (you can describe in general)

- In your opinion, do you think that there is a ***need to introduce*** more number of 'SPs' in a medical program. Give reasons for your answer.

- In your opinion, ***which skills / tasks*** (history taking / clinical examination / counselling etc), are more easier for you to train 'SPs'. Give reasons for your answer.

- In your opinion, ***what are the disciplines*** (internal medicine, surgery, OG etc) that are more easier for you to train 'SPs'. Give reasons for your answer.

- In your opinion, what are the ***advantages*** of introducing 'SPs' in a medical program?

- In your opinion, what are the **difficulties /** ***challenges*** in training 'SPs' for medical program?

- Can you share few ***suggestions***, to overcome the challenges you just mentioned?
