# Supplementary material for: Validating simulated patient programmes in Obstetrics and Gynaecology education: a mixed-method study on training effectiveness and stakeholder perceptions in the GCC
Source: BMC Med Educ. 2025 Oct 17;25:1439. doi: 10.1186/s12909-025-07912-2 (PMC12532415; doi:10.1186/s12909-025-07912-2)
Supplement: Supplementary file 11 — Supplementary Material 11. [file 12909_2025_7912_MOESM11_ESM.pdf]

SP TRAINING SESSIONS

MEDICAL SKILLS AND SIMULATION CENTER (MSSC)

ARABIAN GULF UNIVERSITY

AGENDAS FOR DAY 1 :

- The SPs shall be trained for the main core abilities like Memory, Communication and Professionalism and evaluated for the same.
- SP trainers shall have an individual or group discussion with the SPs explaining the importance of these core abilities and address the concerns from the SPs .
- Duration : 15 mins
- SP trainers shall have a training sessions using a SP script / scenario or a video file to train individual SPs .
- Duration : 30 mins
- Once the training sessions are done then the SPs shall be requested to perform a training and the abilities will be evaluated by the trainers.
- Duration : 30 mins
- A group discussion among the SP trainers and the SPs to address the issues and the methods to rectify the shortcomings .
- Duration : 30 mins
- Finally the Agendas of the Day two training sessions shall be discussed with the SPs before they leave.
- Duration 15 mins
- Total Duration of training sessions 1 : 2 hrs

SIGNATURE: T. Garadah DATE : 07/04/22

(Prof.Taysir Garadah)

SP TRAINING SESSIONS

MEDICAL SKILLS AND SIMULATION CENTER (MSSC)

ARABIAN GULF UNIVERSITY

AGENDAS FOR DAY 2 :

- The SPs shall be trained for the other important abilities related to their performance like , standardization, ability to respond to unexpected questions / situations, emotional control and feedback.
- SP trainers shall have an individual or group discussion with the SPs explaining the importance of the above mentioned subjects and address the concerns from the SPs .
- Duration : 15 mins
- SP trainers shall have a training sessions using a SP script / scenario or a video file to train individual SPs .
- Duration : 30 mins
- Once the training sessions are accomplished, the SPs shall be requested to perform the training and the abilities will be evaluated by the SP trainers and be documented.
- Duration : 30 mins
- A group discussion among the SP trainers and the SPs to address the issues and the methods to rectify the shortcomings .
- Duration : 30 mins
- Finally the Agendas of the Day 2 training sessions shall be discussed with the SPs before they leave.
- Duration 15 mins
- Total Duration of training sessions two : 2 hrs

SIGNATURE: [signature]

(Prof.Taysir Garadah)

DATE : 07/04/22

MEDICAL SKILLS AND SIMULATION CENTER (MSSC)

ARABIAN GULF UNIVERSITY

مركز المحاكاة والمهارات الطبية
Medical Skills and Simulation Center
MSSC

SP (STANDARDIZED PATIENT) TRAINING

Day -1 EVALUATION FORM

NAME :

DATE :

CPR :

NATIONALITY :

MEMORY :<br><br>Remarks:	ATTITUDE:<br>CONFIDANCE :<br><br>Remarks:
COMMUNICATION :<br>English Articulation :<br>Fluency :<br><br>Remarks :	PROFESSIONALISM:<br><br>Remarks:
TOTAL HOURS COMPLETED :	ADDITIONAL REMARKS:

FINAL REMARKS :

1- VERY POOR
2- POOR
3- Average-
4- Good
5- Very Good

ACCEPTED ( )
NOT-ACCEPTED ( )

SIGNATURE : [signature]

07/04/22

MEDICAL SKILLS AND SIMULATION CENTER (MSSC)

ARABIAN GULF UNIVERSITY

مركز المحاكاة والمهارات الطبية
Medical Skills and Simulation Center
MSSC

SP (STANDARDIZED PATIENT) TRAINING

Day -2 EVALUATION FROM

NAME :

DATE :

CPR :

NATIONALITY :

STANDARDIZATION :<br><br>Remarks:	ABILITY TO RESPOND TO UNEXPECTED SITUATIONS:<br><br>Remarks:
EMOTIONAL CONTROL :<br><br>Remarks :	ABILITY TO GIVE FEEDBACK<br><br>Remarks :
TOTAL HOURS COMPLETED:	ADDITIONAL REMARKS:

FINAL REMARKS :

1- VERY POOR
2- POOR
3- Average-
4- Good
5- Very Good

ACCEPTED ( )
NOT-ACCEPTED ( )

SIGNATURE : F. [illegible] 07/04/22
