# Supplementary material for: Validating simulated patient programmes in Obstetrics and Gynaecology education: a mixed-method study on training effectiveness and stakeholder perceptions in the GCC
Source: BMC Med Educ. 2025 Oct 17;25:1439. doi: 10.1186/s12909-025-07912-2 (PMC12532415; doi:10.1186/s12909-025-07912-2)
Supplement: Supplementary file 8 — Supplementary Material 8. [file 12909_2025_7912_MOESM8_ESM.pdf]

MEDICAL SKILLS AND SIMULATION CENTER (MSSC)

ARABIAN GULF UNIVERSITY

مركز المحاكاة والمهارات الطبية
Medical Skills and Simulation Center

SP (STANDARDIZED PATIENT) INTERVIEW

NAME :

AGE / SEX :

CPR :

POST INTERVIEWED FOR :

DATE :

NATIONALITY :

EDUCATIONAL QUALIFICATIONS:<br><br>Remarks:	WORK EXPERIENCE:<br>Current Job:<br><br>Remarks:
COMMUNICATION SKILLS :<br>Languages Known :<br>Fluency :<br><br>Remarks :	GENERAL KNOWLEDGE & ATTITUDE :<br><br>Remarks:
KNOWLEDGE ABOUT SIMULATION:<br><br>Remarks:	PREVIOUS EXPERIENCE AS SP :<br><br>Remarks :
FINAL REMARKS :	

SIGNATURE :
